# Conformational Variability of HIV-1 Env Trimer and Viral Vulnerability

**DOI:** 10.1101/2025.10.09.681376

**Published:** 2025-10-10

**Authors:** Yiwei Cao, Wonpil Im

**Affiliations:** 1 Department of Biological Sciences, Lehigh University, 111 Research Dr, Bethlehem, PA 18015, USA

## Abstract

HIV-1 envelope glycoprotein (Env) is critical for viral fusion and entry into host cells and remains a primary target for vaccine and antiviral drug development. Advances in soluble gp140 trimer design have provided insight into the ectodomain structure and dynamics. However, the membrane-proximal external region (MPER) and transmembrane domain (TMD) are comparatively understudied, and knowledge of the cytoplasmic tail (CT) is virtually absent. Additionally, the ectodomain and TMD have been investigated separately in previous studies. To investigate the trimeric gp120–gp41 as a complete entity and its structural flexibility, we built a full-length model of the gp120–gp41 trimer that is fully glycosylated with N-linked glycans and embedded in a lipid bilayer, and performed all-atom molecular dynamics simulations. Our results show that the ectodomain maintains a rigid internal structure stable in the prefusion state, whereas the intrinsic flexibility of the MPER enables the ectodomain to adopt a range of tilted orientations, potentially enhancing spatial alignment for receptor engagement. The centrally positioned R696 residue in the TMD interacts with lipid headgroups, ions, and the CT residues, resulting in conformational variability in the TMD and perturbations in the surrounding membrane that may facilitate the fusion process. Finally, we demonstrate how simulation trajectories can be leveraged to evaluate the accessibility of antibody epitopes across different regions of the protein.

## Introduction

Human immunodeficiency virus type 1 (HIV-1) is the most prevalent strain of HIV responsible for the development of acquired immunodeficiency syndrome (AIDS). The HIV-1 envelop (Env) consists of a host cell-derived lipid membrane and viral glycoproteins that play a crucial role in mediating viral entry into host cells. The Env glycoprotein is initially synthesized in the endoplasmic reticulum (ER) as a precursor gp160 and cleaved by furin into two subunits, gp120 and gp41. The non-covalently associated gp120–gp41 complex is transported to the cell surface in the form of a trimer, where it is subsequently incorporated into the envelope of nascent virions during viral assembly. The exposure of Env protein is essential for binding to the primary receptor CD4 and the co-receptors CCR5 or CXCR4, triggering membrane fusion and viral entry. However, this exposure also renders the virus susceptible to immune attack. In response to host immune pressure, Env is densely coated with N-linked glycans added during post-translational modification in the ER and Golgi apparatus, which effectively shield vulnerable epitopes from immune recognition.

Since HIV-1 was identified as the cause of AIDS in the early 1980s, extensive research has been conducted to elucidate the mechanisms of viral infection and immune response. A major breakthrough was the design of soluble gp140 trimers, composing gp120 and part of the gp41 subunit. Native-like Env trimers, such as SOSIP,^1^ native flexibly linked (NFL),^2^ and uncleaved prefusion-optimized (UFO) constructs,^3^ mimic the structure of viral spike and serve as valuable antigen targets for developing small molecule inhibitors and broadly neutralizing antibodies (bNAbs). With advances in structure determination techniques, such as X-ray crystallography and cryo-electron microscopy (cryo-EM), numerous high-resolution structures of gp140, both unliganded and antibody-bound, are now available in the Protein Data Bank (PDB). Even with such progresses, however, the remaining portions of gp41, including the membrane-proximal external region (MPER), transmembrane domain (TMD), and cytoplasmic tail (CT), are relatively understudied. The MPER is a highly conserved region targeted by several bNAbs, including 10E8, 2F5, 4E10, and Z13e1.^4–7^ However, in most studies, MPER is examined as a monomeric peptide bound to antibodies or within membrane mimetics such as bicelles and nanodiscs, which do not capture the structure and dynamics of unliganded MPER in the context of the gp120–gp41 trimer embedded in a membrane bilayer. Despite the critical roles of the MPER and TMD in mediating the fusion of viral and host cell membranes,^8,9^ they are often excluded from structural studies due to difficulties in crystallization of hydrophobic TM segments while maintaining their native conformations. Therefore, nuclear magnetic resonance (NMR) spectroscopy remains one of the few viable methods for obtaining structural information of MPER and TMD. However, the NMR studies from different research groups have yielded conflicting conclusions regarding the oligomeric state of the TMD. Reported TMD structures include trimeric coiled coils,^10–13^ monomeric helices,^14^ and trimers that are not tightly bundled.^15^ Similarly, there are different conclusions about the conformation and orientation of MPER. Some studies suggest that gp41 bends at the MPER-TMD boundary (around residue 673), with the entire MPER adopting a helical conformation that lies nearly parallel to the membrane,^11,16^ while others show that the C-terminal residues of MPER forms a continuous helix with the TMD.^10,12,13,17^ In addition, the exceptionally long CT plays an important role in facilitating the incorporation of the Env glycoprotein into virions,^18^ While it is known to contain three conserved amphipathic α-helical segments, referred to as lentiviral lytic peptides (LLPs), proposed models differ in the arrangement of LLPs,^13,19^ and the complete structure of CT and its location in the membrane remains inconclusive.

**Figure 1.**
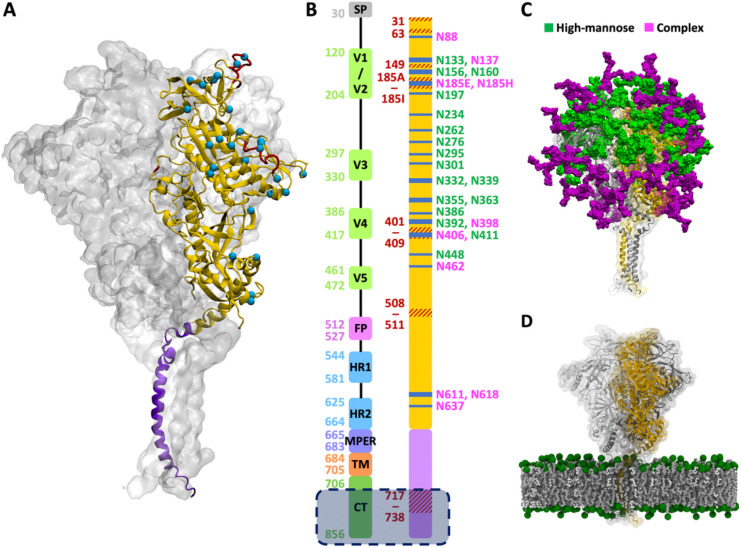
Model structure of a fully glycosylated full-length HIV Env trimer embedded in a membrane. (A) The model structure built by combining the cryo-EM structure of the ectodomain (yellow, PDB ID: 6B0N) with the NMR structure of the MPER and TMD (purple, PDB ID: 7LOI). The missing loops in the PDB structures are highlighted in red, and the glycosylation sites are marked by cyan spheres. (B) Left: assignment of functional domains with boundary residue numbers, including signal peptide (SP), variable regions (V1-V5), fusion peptide (FP), heptad repeats (HR1 and HR2), membrane-proximal external region (MPER), transmembrane domain (TMD), and cytoplasmic tail (CT). Right: missing loops (red) and glycosylation sites (blue). The shaded region at the bottom marks CT residues excluded in the CT-truncated model (see Figure S1 for a model with the full CT included). (C) N-linked glycans with high-mannose (green) and complex (magenta) types. (D) Env trimer embedded in a membrane. Lipid headgroups are highlighted by green spheres and glycans are omitted for visual clarity. Molecular illustrations were prepared using Visual Molecular Dynamics (VMD).^21^

In this work, we built a model of full-length gp120–gp41 trimer embedded in a lipid bilayer mimicking the lipid composition of the mammalian plasma membrane^20^ (Figure 1). We prepared simulation systems varying in the presence of the cleavage site and CT, as well as the initial position of protein in the membrane. Multiple microsecond-long all-atom molecular dynamics (MD) simulations were performed for each system to explore the motions of individual protein domains and the membrane, and to examine how their conformational variability is affected by the difference in the initial configurations. Our results show that the ectodomain undergoes substantial tilting relative to the membrane plane while maintaining a rigid internal structure. In contrast, the MPER and TMD display highly diverse conformations, and their structural variations are influenced by the presence of the CT and the initial TMD position in the membrane. Moreover, we selected several bNAbs targeting the epitopes across different regions of the Env protein and demonstrate that the simulation trajectories can be used to assess the epitope accessibility.

## Results

### The ectodomain maintains a rigid internal structure and tilts independently of the TMD

**Figure 2.**
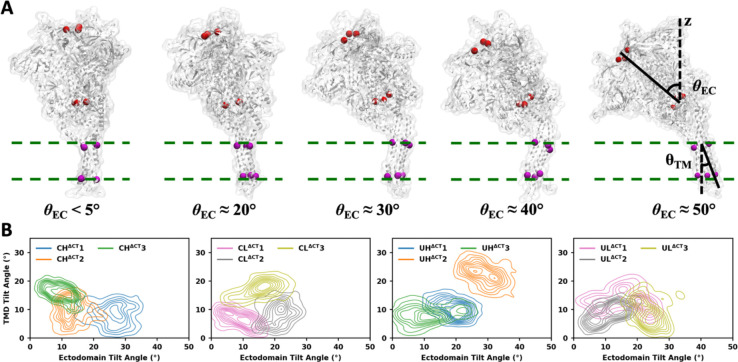
Tilting motions of the ectodomain and TMD are independent. (A) Representative structures illustrating different ectodomain tilt angles and the schematic showing how tilt angles are calculated. (B) Ectodomain tilt versus TMD tilt, calculated from CT-truncated systems with various initial configurations.

As described in the Methods section, multiple simulation systems were constructed, varying in cleavage, TMD positioning, and CT truncation. Simulations trajectories are denoted as CH^CT^1, UL^CT^2, CL^ΔCT^3, UH^ΔCT^3, etc., where the first letter (C/U) indicates cleaved or uncleaved, the second letter (H/L) indicates the high or low initial TMD position, CT/ΔCT indicates the presence or absence of the CT, and the numeric suffix specifies the trajectory index among three replicas (Table S1). In all simulations, both the ectodomain and TMD adopted variable orientations relative to the bilayer plane. To quantify the tilt of two domains, we defined the tilt angles for the ectodomain (*θ*_EC_) and TMD (*θ*_TM_) (Figure 2A). *θ*_EC_ is measured between the bilayer normal and the vector from the center of mass (COM) of G594 (on all three protomers) to the COM of D167, while *θ*_TM_ is measured between the bilayer normal and the vector from the COM of I684 to the COM of V705. Across trajectories, *θ*_EC_ typically ranges from 0° to 40°, with occasional tilting up to 50°. Representative structures for different *θ*_EC_ values are shown in Figure 2A. In contrast to the wide range of *θ*_EC_, *θ*_TM_ generally remained within 0° to 20° with rare excursions to 30° observed in a small fraction of trajectories. We split each 1-μs trajectory into four 0.25-μs intervals, and the variations of *θ*_EC_ and *θ*_TM_ over time show that large conformational changes occurred during the first 0.5 μs, followed by convergence of *θ*_EC_ and *θ*_TM_ distributions during the second 0.5 μs of most trajectories (Figures S2–S5). For CH^ΔCT^, CL^ΔCT^, UH^ΔCT^ and UL^ΔCT^ systems, the *θ*_EC_ and *θ*_TM_ distributions calculated from the second 0.5 μs of three independent trajectories are shown in Figure 2B, and the data for CH^CT^, CL^CT^, UH^CT^ and UL^CT^ systems are shown in Figure S6. Although the combined dataset spans a wide range of *θ*_EC_ (0°–50°) and *θ*_TM_ (0°–30°), each individual trajectory explores only a portion of this space. For instance, CH^ΔCT^1 samples large *θ*_EC_ with small *θ*_TM_, whereas CH^ΔCT^3 samples small *θ*_EC_ with medium-to-large *θ*_TM_. Thus, multiple independent simulations are essential to capture a wide region of the conformational space. Most importantly, no consistent correlation was observed between *θ*_EC_ and *θ*_TM_, either within single trajectories or across all trajectories combined. Pearson correlation coefficients of *θ*_EC_ and *θ*_TM_ in single trajectories varies between −0.5 and 0.5, with examples such as CH^ΔCT^1 and CH^ΔCT^2 showing similar *θ*_TM_ but distinct θ_EC_ values, and CL^ΔCT^1 and CL^ΔCT^3 showing similar θ_EC_ but distinct *θ*_TM_ values.

**Figure 3.**
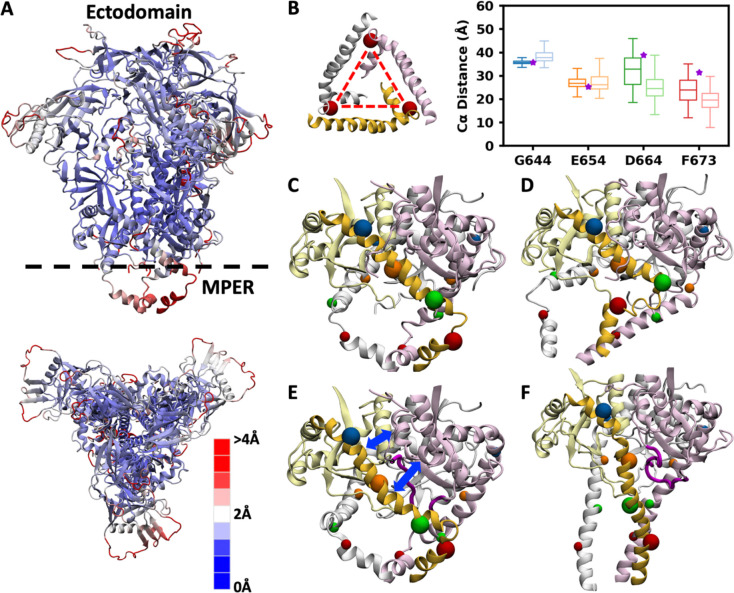
Ectodomain is rigid, whereas the MPER is highly flexible and adopts diverse conformations. (A) Top and side views of the ectodomain and MPER in the cleaved system, with RMSF indicated by color. (B) Schematic illustrating the calculation of interchain distance and its distributions measured at the Cα atoms of G644, E654, D664, and F673. Cleaved and uncleaved systems are represented by solid and transparent colors, respectively. The initial values of interchain distances are marked by purple stars. (C–F) Local structures of the ectodomain C-terminus and MPER. The HR2 helix and MPER in one protomer are highlighted in dark yellow, with the Cα atoms of four selected residues marked by blue, orange, green and red spheres. (C) The initial conformation and (D) representative snapshot from simulations of the cleaved system. (E) The initial conformation and (F) representative snapshot from simulations of the uncleaved system.

Despite the considerable tilting of the ectodomain relative to the membrane, its internal structure remains rigid and well-preserved throughout the simulations. The root-mean-square fluctuation (RMSF) and root-mean-square deviation (RMSD) of the ectodomain were calculated after the snapshots from each trajectory were aligned to the initial structure by maximizing the overlap in the ectodomain. The majority of the ectodomain displays low RMSF (< 2 Å), and the RMSD stabilized around 4 Å after an initial rise during the first 250 ns (Figures 3A and S7). Higher RMSF values were observed in the residues missing from the cryo-EM structure highlighted in red in Figures 1A and B, and in part of HR1 (Q551-H564) that forms a flexible loop at the interface between two neighboring protomers. In addition, the RMSF of the MPER was also calculated with the trajectories aligned by the ectodomain. The entire MPER (K665–R683) and the adjacent HR2 segment (L660–D664) at the C-terminus of the ectodomain exhibited elevated fluctuations. The gp120–gp41 model was built based on the NMR structure in which the MPER adopts a bent conformation consisting of two helices joined by a sharp turn. The resulting trimer widens from the HR2 helix to the midpoint of the MPER (F673) and narrows from F673 to the TMD. However, this specific conformation was not maintained throughout the simulations. We measured the inter-chain distances between the Cα atoms of corresponding residues (G644, E654, D664, and F673) on neighboring protomers to characterize the structural variation along the HR2 helix and the MPER (Figure 3B). The inter-chain distances of G644 and E654 maintained narrow distributions centered on their initial values, while those of D664 and F673 exhibited broader distributions, reflecting inward shifts of three protomers. This effect was more pronounced in uncleaved systems. In cleaved systems, the HR2 helix interacted with residues M530-N543 and L619-N625 of neighboring protomers, which helps to stabilize the bent MPER conformation (Figures 3C and D). However, these interactions were not consistently observed across all protomers and all simulations, and therefore we still observed considerable conformational variability in this region. In uncleaved systems, the closed loop at the cleavage site occupied the space between the HR2 helix and the neighboring protomer, disrupting their interactions and facilitating inward shifts of the HR2 helix (Figures 3E and F).

### The energetically unfavorable R696 in the hydrophobic core results in asymmetric, kinked TMD conformations and disrupts membrane integrity

**Figure 4.**
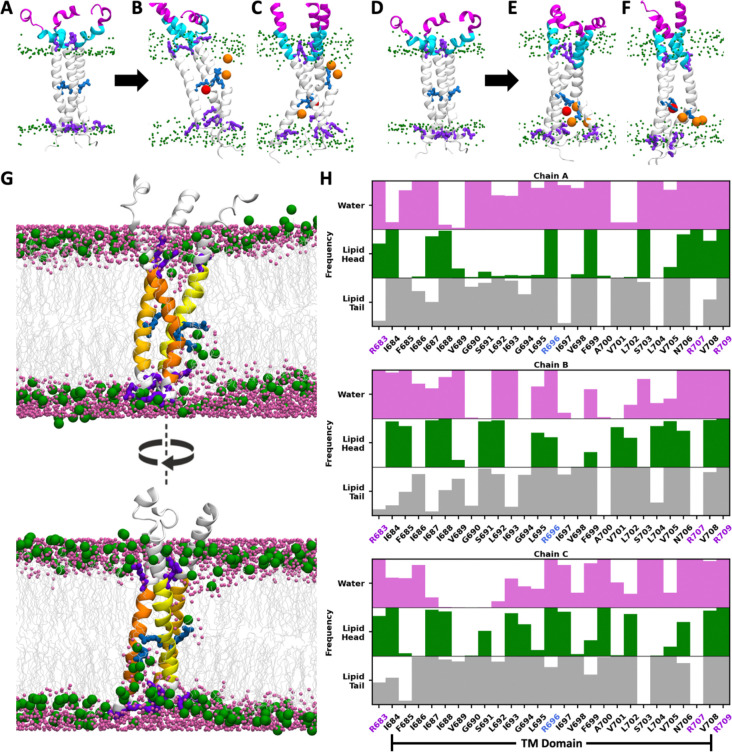
R696 interacts with lipid headgroups and disrupts membrane integrity. (A–C) MPER and TMD in the CT-truncated system with the “high” TMD configuration. MPER-N, MPER-C, and TMD are shown in magenta, cyan, and white, respectively. Lipid headgroups, R696, and the residues anchored in the lipid headgroups (R683, R707 and R709) are shown in green, blue, and purple, respectively. Lipid headgroups and ions interacting with R696 are highlighted in orange and red, respectively. (A) Initial conformation. (B and C) Representative snapshots from different trajectories. (D–F) MPER and TMD in the CT-truncated system with the “low” TMD configuration. (G) Two side views of the same snapshot where R696 of one protomer interacts with lipid headgroups in the exoplasmic leaflet and R696 of two protomers interact with lipid headgroups in the cytoplasmic leaflet. Lipid headgroups and tails are shown in green and gray, and water molecules in magenta. TMD of three protomers (i.e., chains A, B and C) are shown in light yellow, dark yellow and orange, respectively. (H) Frequency of TMD residues interacting with lipid headgroups, lipid tails, and water. Bar shading reflects interaction frequency, from fully filled (100%) to unfilled (0%).

Unlike the predominantly hydrophobic TMDs commonly found in many viral envelop proteins, the gp41 TMD contains multiple charged residues: R683 at the N-terminal boundary, R707 and R709 at the C-terminal boundary, and a central arginine, R696 (Figure 4A). In simulations, R683 consistently interacted with lipid headgroups in the exoplasmic leaflet, while R707 and R709 interacted with those in the cytoplasmic leaflet, together acting as anchors that secure the TMD within the bilayer. In the cryo-EM structure (PDB ID: 7LOI), the side chain of R696 forms cation-π interaction with the side chain of F699 and a hydrogen bond with the backbone carbonyl group of L692, but it is oriented outward from the helical bundle. When embedded in the bilayer, however, this configuration is energetically unfavorable, as the positively charged side chain directly contacts hydrophobic lipid tails.^22^ Early in the simulations, the TMD rapidly rearranged to allow R696 to interact with more favorable partners, including negatively charged lipid headgroups from either leaflet, ions and water molecules diffusing into the bilayer center, as well as the polar and positively charged groups in the CT when it is present. Because the limited space at the TMD core can only accommodate at most two inward-facing arginine residues, at least one R696 is forced outward to interact with lipid headgroups or CT residues. The differences in arginine orientation and interacting partners give rise to asymmetric protomer conformations and distinct TMD tilts (Figures 4A–F). When R696 points outward, its interactions with lipid head groups or CT residues can be strong enough to destabilize the local helix, introducing a kink into the TMD. Representative snapshots from different trajectories illustrate these asymmetric kinked conformations (Figure S8–S15). Beyond local deformation of the TMD, R696–lipid interactions perturb bilayer organization, inducing the translocation of lipid headgroups and water molecules toward the bilayer center (Figure 4G). We calculated the interaction frequencies of each TMD residue with lipid headgroups, lipid tails, and water molecules. The results show that the membrane disruption is persistent since many non-terminal TMD residues maintain frequent contacts with water and lipid headgroups throughout the simulation (Figure 4H).

To explore whether R696 exhibits a preference for the exoplasmic versus cytoplasmic leaflet, or interacts with either randomly, we generated two initial structures (high and low) with the TMD positioned at two distinct positions, separated by 4 Å along the membrane normal (see Methods). In simulations initiated from the “high” TMD configuration, R696 residues in three protomers interacted with the lipid headgroups in either leaflet (Figures 4B, C, S8 and S10) In contrast, in simulations initiated from the “low” TMD configuration, R696 residues interacted exclusively with the headgroups in the cytoplasmic leaflet (Figures 4E, F, S9 and S11). In the full-length systems, the plate-shaped CT occupies substantial space in the cytoplasmic leaflet, displacing lipids during model construction. Because the CT is not thick enough to fully span the cytoplasmic leaflet, an empty gap remained between the CT and the exoplasmic leaflet (Figure S1D). Over time, lipids in the exoplasmic leaflet shifted downward while the CT residues moved upward to fill this space, causing local bilayer thinning. Under these conditions, upward-oriented R696 can still interact with the headgroups in the exoplasmic leaflet, but downward-oriented R696 primarily contacted CT residues, with rare cases of lipids migrating upward from the cytoplasmic leaflet and approaching the protein. A comparison of all full-length systems (Figures S12–S15) shows that R696 preferentially adopted downward orientations in the simulations initiated from the “low” TMD configuration (Figures S13 and S15). In these cases, upward-shifted CT residues interact with the C-terminal half of the TMD, rather than solely with R696, resulting in deeper burial of the TMD in the membrane.

### MPER adopts diverse conformations, and its exposure depends on both MPER and TMD conformations

Starting from the initial helix-turn-helix conformation consisting of two separate helical segments, the N-terminal half (MPER-N) and the C-terminal half (MPER-C), MPER underwent rapid rearrangements, and a wide variety of conformations were sampled across all trajectories. In the initial structure, the trimeric MPER was positioned perpendicular to the membrane, with the helical MPER-N tilted inward and MPER-C tilted outward (Figure 4A). Such conformation and orientation were maintained in some trajectories such as CL^ΔCT^3 (Figure S9C), but in others the helix-turn-helix MPER shifted into a horizontal orientation parallel to the membrane surface (Figure S13A) or a more vertical arrangement with both MPER-N and MPER-C tilted outward (Figures 4E and S111A). It was also observed that the HR2 helix in the ectodomain, MPER, and TMD merged into a continuous long helix (Figures 4C, F, and S10C). In addition, it was common for part of the MPER, particularly the MPER-C, to lose its helical structure and become a random coil. Therefore, the distinct MPER conformations reported in various experimental studies can be all valid as each captures a possible state within the highly flexible conformational landscape of the MPER. It is noteworthy that the MPER in three protomers can adopt different conformations and orientations, resulting in asymmetric local structures.

**Figure 5.**
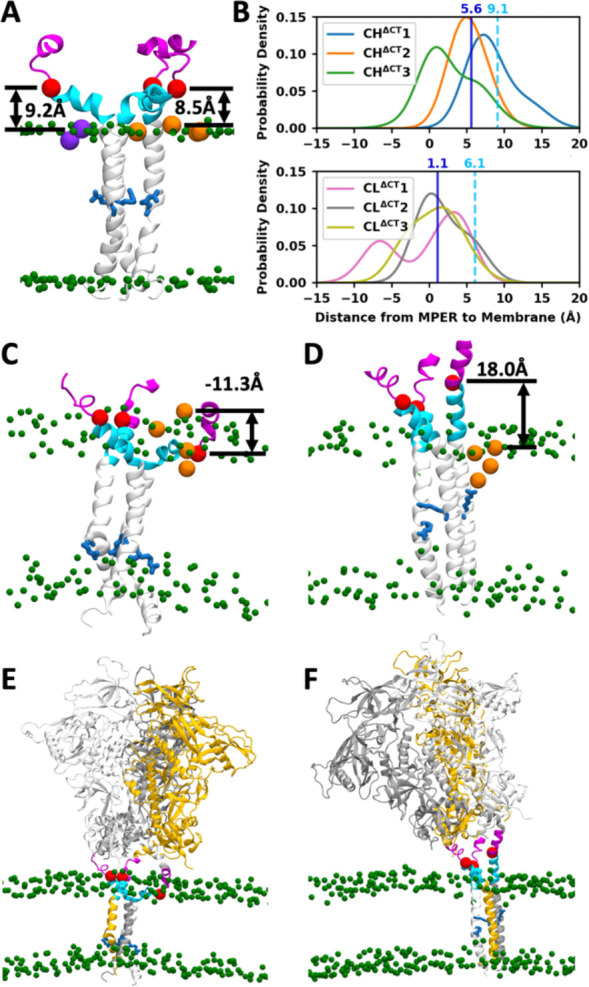
MPER exhibits diverse conformations, and its exposure depends on both MPER and TMD. (A) The initial structure of the CH^ΔCT^ system, where *d*_F673_ of two promoters equals 8.5 Å and 9.2 Å. Lipid headgroups are shown in green and R696 in blue. *d*_F673_ is defined as the distance from the Cɑ of F673 (red) to the highest among the adjacent lipid headgroups (orange and purple). (B) Distribution of *d*_F673_ in the CL^ΔCT^ and CH^ΔCT^ systems. The cyan dashed line indicates the mean *d*_F673_ of three protomers in the initial structure, and the blue solid line indicates the mean across all data sampled from simulations. (C and D) Representative snapshots illustrating the buried (C) and exposed (D) MPER. (E and F) The entire trimer structures corresponding to (C) and (D), respectively.

Because the MPER is a target of multiple bNAbs, we next examined how its exposure is affected by TMD conformation. As described above, interactions between R696 and lipid headgroups affect the burial depth of the TMD, thereby influencing the positioning of the adjacent MPER. To quantify MPER exposure, we measured the vertical distance from the Cɑ of F673, approximately the midpoint of MPER, to the highest point of the neighboring lipid headgroups, denoted by *d*_F673_. Positive values indicate that F673 lies above the bilayer surface, and negative values indicate its membrane burial. In the initial ‘low’ and ‘high’ TMD configurations, *d*_F673_ was 6.1 Å and 9.1 Å, respectively, but across simulations it spanned a wide range from −15 Å to 20 Å (Figures 5A and B). Two examples illustrate this variability. In the first example, all three R696 residues interacted with the cytoplasmic leaflet, drawing the TMD deeper into the membrane. Consequently, the entire MPER-C and most of MPER-N were buried in the membrane, with one F673 positioned 11.3 Å below the membrane surface, thus bringing the ectodomain in close proximity to the membrane (Figures 5C and E). In the second example, two R696 residues interacted with the exoplasmic leaflet, while one interacted with the cytoplasmic leaflet. In the protomer with upward-oriented R696, the MPER-N, MPER-C, and TMD formed a continuous helix. As a result, most of the MPER extended outside the bilayer, with *d*_F673_ reaching 18.0 Å and the ectodomain displaced farther from the membrane (Figures 5D and F). The *d*_F673_ distributions across all cleaved CT-truncated systems suggest that *d*_F673_ tends to be smaller when the simulations started from the “low” TMD configuration (Figure 5B). In both “high” and “low” configurations, the mean *d*_F673_ calculated from the simulation trajectories is smaller than its value calculated from the initial structure, due to both protein conformational change and lipid diffusion. In the “low” configuration, *d*_F673_ decreased by 5.0 Å (from 6.1 Å to 1.1 Å), a slightly larger reduction than in the “high” configuration where *d*_F673_ dropped by 3.5 Å (from 9.1 Å to 5.6 Å), indicating that deeper MPER burial arises from a larger-scale protein motion rather than merely from a lower initial placement.

### Ectodomain epitopes are conditionally accessible, whereas MPER epitopes are virtually inaccessible in the closed prefusion state

**Figure 6.**
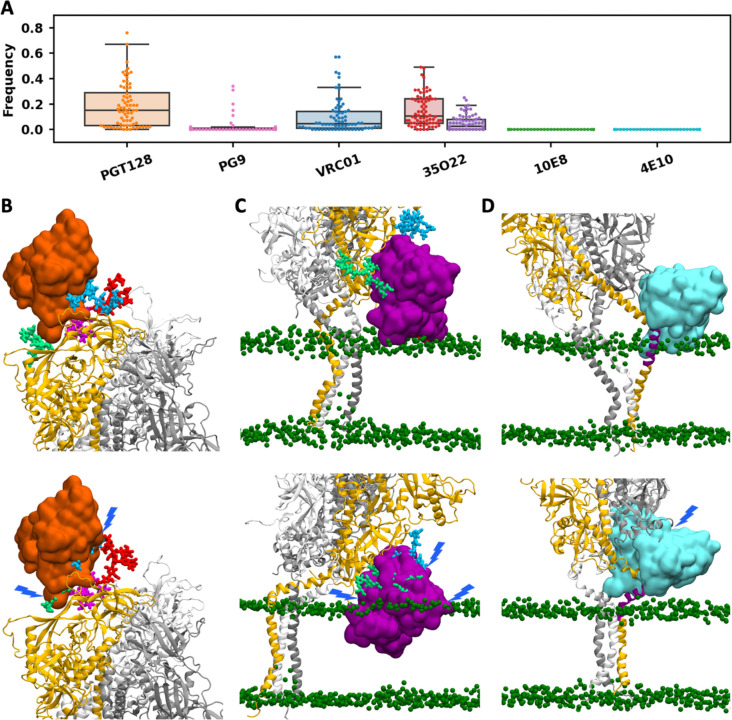
Antibody epitope accessibility. (A) The frequency of accessibility. Each marker represents the epitope on one of the three protomers across all trajectories. For 35O22, red indicates the accessibility frequency without considering steric clashes with the membrane, while purple indicates the frequency accounting for clashes with the membrane. (B-D) Representative snapshots showing conformations with the epitope exposed (upper) and shielded (lower) for antibodies PGT128, 35O22, and 4E10, respectively. The antibody V_H_ and V_L_ domains are shown in surface representation, with lipid head groups in green spheres and glycans that may interfere with the antibody in distinct colors.

To access the accessibility of epitopes on different parts of the trimeric Env protein in the prefusion state, we quantified how frequent their epitopes were exposed without steric clashes from neighboring protein residues, glycans or membrane lipids to six selected antibodies: PGT128 targeting the V3 loop and the N332 glycan (PDB ID: 5JSA); PG9 targeting the V1/V2 loop (PDB ID: 3U2S); VRC01 targeting the CD4 binding site (PDB ID: 4LST); 35O22 targeting the gp120–gp41 interface (PDB ID: 4TVP); and 10E8 (PDB ID: 6VPX) and 4E10 (PDB ID: 1TZG) targeting the MPER (Figure 6A, Table S2–S5).

The epitope of PGT128 consists of the V3 loop and N332 glycan, and it is widely recognized that PGT128 binding is mediated by the protein-protein interaction with V3 loop and the protein-glycan interaction with N332 glycan, facilitated by its extra-long HCDR3 loop penetrating the glycan shield (Figure S16). Due to the absence of defined secondary structures, glycans exhibit greater conformational flexibility than the protein, although the crowded surface of gp120–gp41 may partially restrict the motion of glycans. N332 glycan must adopt specific conformations to enable specific interactions with PGT128, whereas most conformations block antibody approach. Additional glycans on N137, N156, and N301 can further occlude the site (Figure 6B). Epitope accessibility to PGT128 varied widely across protomers and trajectories, with many cases exceeding 35%. In about half trajectories, at least one protomer exhibited >35% accessibility (Table S2). For instance, in system CH^ΔCT^1, two protomers showed accessibility >40%, whereas in system CL^CT^2, all three protomers are <5%. The second antibody, PG9, targets the V1/V2 apex, where binding can be hindered by six glycans, N156, N160, and N185E on the same protomer, and N160, N185E, and N185H on the neighboring protomer (Figure S17). In most trajectories, the epitopes on all three protomers were occluded in >95% of the snapshots, with the exception of a few systems (CH^ΔCT^1, CH^ΔCT^2, and CL^ΔCT^3) where accessibility was non-negligible. (Table S3). The third antibody, VRC01, targets the CD4 binding site where six glycans, N185H, N197, N276, N363, and N462 on the same protomer and N301 on the neighboring protomer, can interfere with antibody binding (Figure S18). Similar to PGT128, the VRC01 epitope is moderately to highly accessible to at least one protomer in many trajectories, whereas in some trajectories, the epitopes on all three protomers are nearly completely shielded (Table S4).

35O22 targets the gp120–gp41 interface that is not intrinsically membrane-proximal. However, its binding orientation combined with ectodomain tilting can lead to clashes of 35O22 with the membrane (Figure S19). Comparing accessibility with and without including the steric effects of membrane lipids shows that the membrane can hinder 35O22 binding, particularly when it approaches from the direction of ectodomain tilt (Figure 6B). For example, in system UH^ΔCT^2, the accessibility frequencies of the three protomers were 49%, 5%, and 25%, when only the shielding of three glycans (N88, N234, and N618) was considered. When the steric effects of both glycans and membrane lipids were included, the first protomer’s frequency dropped from 49% to 1%, while the other two remain essentially unchanged (Table S5).

The last two antibodies, 10E8 and 4E10, target the MPER. In the PDB structures of 10E8, the epitope is a 17-residue peptide corresponding to residues 671–687 in this work. In the PDB structure of 4E10, the epitope is a 12-residue peptide corresponding to residues 669–680. Superposition of these antibodies onto the initial simulation structure revealed extensive clashes with the protein and glycans linked to N611, N618, and N637 on the neighboring protomer, as well as slight steric clashes with the membrane (Figures S20 and S21). Using the strict criteria (≤10 heavy-atom clashes with protein/glycan and ≤20 with lipids), no snapshot was identified in which the epitope of either antibody was accessible. Even with relaxed criteria (≤20 heavy-atom clashes with protein/glycan or ≤40 with lipids), accessible cases remain rare, which require either large ectodomain tilts to create space on the opposite side or the MPER transitioning into continuous helices to displace the ectodomain from the membrane (Figures 6C, S22–S25). Given the extremely low frequency of such specific conformations, MPER epitopes are effectively inaccessible in the prefusion trimer, suggesting that MPER-targeting antibodies such as 10E8 and 4E10 act at later stages of viral entry.

## Discussion

The hydrophobic and flexible nature of the MPER has made it difficult to crystallize, and most structural insights have come from NMR studies. However, structural information on the MPER and TMD has been inconsistent in the literature. For example, one study of the MPER in DPC micelles suggested that the MPER adopted a distorted helical structure lying parallel to the membrane surface,^16^ while NMR studies using gp41 fragments containing part of the MPER and TMD embedded in bicelles or bilayers reached conflicting conclusions. Some proposed a kink^14^ or turn^11^ between the MPER and TMD, whereas others suggested that the MPER and TMD formed a continuous helix aligned perpendicular to the membrane.^10,17^ In the NMR structure (PDB ID: 7LOI) used to build our model structure, a kink appears between the MPER and TMD, along with a turn in the middle of the MPER.^13,23^ Crystal structures of MPER peptides bound to various antibodies show that in addition to the helix-turn-helix conformation, MPER can also adopt alternative structures in which the MPER-C remains helical while part of the MPER-N becomes unstructured.^24^ Such variability underscores the intrinsic structural flexibility of the MPER, with each experimental method capturing only a subset of possible states under specific conditions. In this study, we employed MD simulations to sample a broader conformational landscape and gain a more comprehensive view of the structural heterogeneity of the MPER. The results reveal that the MPER can adopt all of the conformations reported experimentally. While MPER plasticity has been linked to its role in virus-host membrane fusion because it enables the ectodomain and TMD to adopt distinct orientations during large-scale structural rearrangements, our results show that this flexibility is already present in the prefusion state. At this stage, the MPER functions as a flexible hinge that facilitates ectodomain tilting, which can be critical for the spatial alignment of the CD4-binding site with the host receptor for efficient receptor engagement.

To probe antibody recognition, we assessed epitope accessibility using snapshots extracted from simulation trajectories. Unlike static experimental structures, this approach incorporates protein dynamics as well as the influence of glycans and membrane lipids, yielding a frequency-based measure indicating how often each epitope is accessible to its corresponding antibody, rather than a binary accessible/occluded classification. It is worth noting that viral glycans are highly variable in both site occupancy and the specific glycoform present at each glycosylation site, as indicated by mass spectrometry data.^25,26^ For structure modeling, however, we assumed full occupancy at all glycosylation sites and selected a single representative glycoform per site. As a result, the estimated glycan shield may not fully reflect the in vivo situation, and the calculated frequency of antibody accessibility should be regarded as an approximation. Our results indicate that epitopes on the ectodomain, though heavily shielded by glycans, can still become transiently accessible in the closed prefusion state. The frequency provides a quantitative measurement of how vulnerable each epitope is to antibody binding. When estimating steric clashes between the membrane and antibodies, we adopted relaxed cutoffs to account for lipid fluidity, but the membrane due to fast lipid dynamics may in reality accommodate even greater steric overlap. Although the MPER can transiently extend out of the membrane when gp120–gp41 remains in the prefusion state, MPER-targeting antibodies still encounter substantial steric hindrance from the bulky gp120 subunit, surrounding glycans, and the membrane throughout nearly all simulations. Therefore, these antibodies are unlikely to adopt the proper orientations required to bind their epitopes. This is consistent with experiment studies indicating that MPER-targeting antibodies bind effectively only after the gp120–gp41 trimer undergoes major conformational rearrangements toward a fusion-intermediate or post-fusion state.^27–29^

Overall, the data presented here demonstrate that structural modeling integrated with molecular dynamic simulations can be applied to complex biomolecular systems, such as the gp120–gp41 trimer embedded in a membrane bilayer, which enables detailed characterization of protein dynamics at the molecular level and facilitates the investigation of antigen-antibody interactions, thereby offering potential guidance for rational vaccine design.

## Methods

### System building

#### Modeling of full-length gp120–gp41 trimer

The full-length gp120–gp41 trimeric model was generated by combining the crystal structure of the ectodomain with the NMR structure containing the MPER, TMD, and CT (Figure 1A and S1A). Considering structure resolution and the number of missing residues, we selected the crystal structure of a soluble Env trimer exhibiting a closed-from, native-like prefusion conformation (PDB ID: 6B0N),^30^ which contains the entire ectodomain including the V1-V5 loops, fusion peptide (FP), heptad repeat 1 (HR1), and heptad repeat 2 (HR2) (Figure 1B). In contrast to the ectodomain well represented in the PDB with numerous crystal and cryo-EM structures, structural data for the TMD are scarce. For this region, we chose the NMR structure (PDB ID: 7LOI)^13^ containing the MPER, TMD, and CT. Both 6B0N and 7LOI are trimeric structures and the C-terminus of 6B0N includes five residues (L660–D664) overlapping with the N-terminus of 7LOI. Measurement of the Cα–Cα distances for D664 in the three protomers revealed that the inter-protomer spacing in 7LOI (16 Å) was much shorter than in 6B0N (39 Å) (Figure S26A). To reconcile this difference, we performed short MD simulations on 6B0N with residues E32–Q640 fixed and a distance restraint applied to D664 in order to reduce the Cα–Cα distance to 33 Å. Similarly, for 7LOI, residues I675–L856 were fixed, and a distance restraint was applied to D664 to increase the distance to 33 Å (Figure S26B). The two adjusted structures were then combined by aligning the overlapping residues. The missing loops in the ectodomain (T63, D149, E185A–N185I, S401–G409) and the unresolved region between the TM and CT (F717–G738) were grafted from a modeled structure generated by I-TASSER^31^ (Figure S26C). 6B0N is a native flexibly linked (NFL) trimer in which the furin cleavage site ^508^REKR^511^ is substituted with a 10-residue linker ^508^GGGGSGGGGS^511^, but the underlined residues are unresolved in the structure. To construct the cleaved gp120–gp41 model, we removed the initial “GG” and final “S” from 6B0N. For the uncleaved gp160 model, we closed the cleaved loop by adding ^508^REKR^511^ back to the structure. If we simply connect G507 and A512 without adjusting the conformation of flanking loops on both sides of the cleavage site, two neighboring protomers become entangled, yielding a knot-like fold (Figures S27A and B). In 6B0N, the helical HR2 region lies at the C-terminus of each protomer, allowing it to thread through a loop formed by the neighboring protomer (Figure S27C). However, in the context of the full-length gp120–gp41, it is implausible that the three protomers are entangled as it does not conform to a realistic folding pathway. Therefore, we performed short MD simulations with restraints to adjust the conformations of the flanking loops and the HR2 helix before closing the loop with REKR fragment (Figures S27D and E). Two mutations (S764C and S837C) were applied to CT to reintroduce the palmitoylation sites, and lipid tails oriented towards the hydrophobic core of the bilayer were added to the palmitoylation sites (Figure S1D). In addition to the full-length model, we built the CT-truncated model by removing the modeled residues F717–G738 that are originally unresolved in the PDB structure 7LOI, and the residues E739–L856 that form the large CT plate.

#### Glycosylation

N-linked glycans were modeled using *Glycan Reader & Modeler*^32–34^ in CHARMM-GUI.^35^ The reported mass spectrometry (MS) experiments have revealed the probability of different types of N-linked glycans at each glycosylation site.^25,26^ For each site, we selected the one with the highest probability. As the MS data provide only the glycan type (high-mannose, hybrid, and complex) and composition (number of each monosaccharide unit), a representative isomer was chosen when multiple isomers corresponded to the same composition. For example, HexNAc(2)Hex(9), i.e., the Man_9_ glycan, has a single isomer, whereas HexNAc(2)Hex(8), i.e., the Man_8_ glycan, has multiple isomers depending on which non-reducing terminal mannose is trimmed from Man_9_. For complex N-linked glycans, one composition can correspond to multiple isomers differing in the number of branches on the α1–3 and α1–6 arms and in the placement of non-reducing terminal neuraminic acid (Neu5Ac). In the absence of linkage-specific information, one isomer was arbitrarily selected in cases of multiple possibilities. The selected glycan sequences for the 27 glycosylation sites in each promoter are summarized in Table S6.

#### Membrane

The full-length and CT-truncated gp120–gp41 models were embedded into an asymmetric lipid bilayer with the lipid composition corresponding to a mammalian plasma membrane,^20^ which consists of phosphatidylcholine (PC), phosphatidylethanolamine (PE), phosphatidylinositol (PI), phosphatidylserine (PS), phosphatidic acid (PA), sphingomyelin (SM), cholesterol (CHOL), and glucosylceramide (GlcCer). In the CT-truncated system, the exoplasmic and cytoplasmic leaflets contain similar numbers of lipids, although their compositions differ. In the full-length system, the cytoplasmic leaflet contains approximately 100 fewer lipids than the exoplasmic leaflet due to the space occupied by the CT. The lipid composition is summarized in Table S7. Although it is recognized that the charged residues R683, R707, and R709, flanking the N- and C-termini of the TMD, are anchored within the lipid headgroups, there remains some flexibility to shift the TMD slightly up or down within the membrane. Assuming the lipid bilayer is aligned parallel to the xy-plane, the protein was positioned at two distinct depths along the z-axis, differing by approximately 4 Å, referred to as the “high” and “low” TMD configurations. To examine whether this subtle difference in the initial configurations leads to distinct conformational changes during simulations, we initiated independent simulations from each configuration. The glycoprotein-membrane system was solvated in a box of approximately 210 × 210 × 260 Å^3^ using the TIP3P water model,^36^ and KCl was added at a concentration of 0.15 M to neutralize the system. Final simulation input files were generated using *Membrane Builder*^37–41^ in CHARMM-GUI.

#### Simulation Details

The combination of cleaved vs. uncleaved, full-length vs. CT-truncated, and high vs. low TMD positions in the membrane results in eight distinct configurations, and we performed three independent 1-μs all-atom MD simulations for each configuration. The CHARMM36(m) force field^42–46^ was used for proteins, carbohydrates, and lipids. The total number of atoms is approximately 1,100,000 (including ~300,000 water molecules and ~1,700 ions), with slight variations between systems. van der Waals interactions were smoothly switched off over 10–12 Å using a force-based switching function,^47^ and long-range electrostatic interactions were calculated using the particle-mesh Ewald method^48^ with a mesh size of ~1 Å. All simulations were performed using GROMACS.^49^ Bond lengths and angles involving hydrogens were constrained using the LINCS algorithm.^50^ The system was equilibrated in the canonical (NVT) ensemble at 310.15 K for 2 × 1.25 ns with a 1-fs time step using the Berendsen thermostat^51^ (coupling constant *τ*_t_ = 1 ps), and then switched to the isothermal–isobaric (NPT) ensemble for 1.25 ns with a 1-fs time step, followed by 3 × 5 ns with a 2-fs time step using the Berendsen thermostat (*τ*_t_ = 1 ps) and barostat (reference pressure = 1 bar, coupling constant *τ*_p_ = 5 ps, compressibility = 4.5×10^−5^ bar^−1^). Positional and dihedral restraints were applied to proteins, glycans, and lipids, with force constants progressively reduced over successive intervals. In the production run, temperature was maintained using the Nosé–Hoover thermostat^52,53^ and the pressure coupling was applied using the semi-isotropic Parrinello–Rahman barostat.^54,55^ A 4-fs time step was used with the hydrogen mass repartitioning technique^56^ and all restraint potentials were removed. The Python library MDTraj^57^ was used to analyze the simulation trajectories.

#### Assessment of Antibody Epitope Accessibility

We selected six antibodies based on epitope location and the availability of PDB structures containing both antibody and epitope: PGT128 targeting the V3 loop and the N332 glycan (PDB ID: 5JSA);^3^ PG9 targeting the V1/V2 loop (PDB ID: 3U2S);^58^ VRC01 targeting the CD4 binding site (PDB ID: 4LST);^59^ 35O22 targeting the gp120–gp41 interface (PDB ID: 4TVP);^60^ 10E8 (PDB ID: 6VPX)^61^ and 4E10 (PDB ID: 1TZG)^6^ targeting the MPER. For each antibody–epitope complex, TM-align^62^ was used to identify the optimal alignment between the epitope from the PDB structure and each protomer of the trimeric protein, and the resulting rotation matrix was applied to place the antibody relative to each protomer. Steric clashes were then assessed by counting any heavy atom of protein, glycan, or lipid within 2 Å of a heavy atom of the antibody. An epitope was considered occluded by protein and glycans if more than 10 heavy-atom clashes occurred with these components. To account for membrane flexibility, we adopted a more permissive cutoff for lipids: the epitope was classified as occluded by the membrane if more than 20 lipid heavy-atom clashes were detected. To calculate the frequency of epitope accessibility, snapshots were extracted from simulation trajectories, and structural alignment and assessment of steric clashes were performed on each snapshot.

## Supplementary Material

Supplement 1

## Data Availability

The modeled structures, force field parameters, and GROMACS input files are available on Zenodo [https://doi.org/10.5281/zenodo.17259909].

## Highlights

- Structural modeling and molecular dynamics simulation of fully glycosylated, full-length gp120–gp41 embedded in a lipid bilayer mimicking a realistic cell membrane.
- The ectodomain adopts a wide range of tilt angles while maintaining internal structural rigidity.
- The charged midpoint residue of the transmembrane domain (TMD), R696, perturbs the lipid bilayer and induces distortions in the TMD helices.
- The highly flexible membrane proximal-external region (MPER) adopts diverse conformations, allowing independent motion of the ectodomain and TMD.
- In silico antibody accessibility analysis indicates that ectodomain epitopes are transiently exposed, while MPER epitopes are virtually always occluded in the pre-fusion trimer.

## Code availability

The code used in this article can be found at https://doi.org/10.5281/zenodo.17259951

## References

[1] Sanders R. W. A next-generation cleaved, soluble HIV-1 Env trimer, BG505 SOSIP.664 gp140, expresses multiple epitopes for broadly neutralizing but not non-neutralizing antibodies. PLoS Pathog. 9, e1003618 (2013). 24068931 10.1371/journal.ppat.1003618 PMC3777863

[2] Sharma S. K. Cleavage-independent HIV-1 Env trimers engineered as soluble native spike mimetics for vaccine design. Cell Rep. 11, 539–550 (2015). 25892233 10.1016/j.celrep.2015.03.047 PMC4637274

[3] Kong L. Uncleaved prefusion-optimized gp140 trimers derived from analysis of HIV-1 envelope metastability. Nat. Commun. 7, 12040 (2016). 27349805 10.1038/ncomms12040 PMC4931249

[4] Huang J. Broad and potent neutralization of HIV-1 by a gp41-specific human antibody. Nature 491, 406–412 (2012). 23151583 10.1038/nature11544 PMC4854285

[5] Ofek G. Structure and mechanistic analysis of the anti-human immunodeficiency virus type 1 antibody 2F5 in complex with its gp41 epitope. J. Virol. 78, 10724–10737 (2004). 15367639 10.1128/JVI.78.19.10724-10737.2004 PMC516390

[6] Cardoso R. M. Broadly neutralizing anti-HIV antibody 4E10 recognizes a helical conformation of a highly conserved fusion-associated motif in gp41. Immunity 22, 163–173 (2005). 15723805 10.1016/j.immuni.2004.12.011

[7] Pejchal R. A conformational switch in human immunodeficiency virus gp41 revealed by the structures of overlapping epitopes recognized by neutralizing antibodies. J. Virol. 83, 8451–8462 (2009). 19515770 10.1128/JVI.00685-09 PMC2738203

[8] Salzwedel K., West J. T. & Hunter E. A conserved tryptophan-rich motif in the membrane-proximal region of the human immunodeficiency virus type 1 gp41 ectodomain is important for Env-mediated fusion and virus infectivity. J. Virol. 73, 2469–2480 (1999). 9971832 10.1128/jvi.73.3.2469-2480.1999 PMC104494

[9] Miyauchi K. Role of the specific amino acid sequence of the membrane-spanning domain of human immunodeficiency virus type 1 in membrane fusion. J. Virol. 79, 4720–4729 (2005). 15795258 10.1128/JVI.79.8.4720-4729.2005 PMC1069530

[10] Dev J. Structural basis for membrane anchoring of HIV-1 envelope spike. Science 353, 172–175 (2016). 27338706 10.1126/science.aaf7066 PMC5085267

[11] Kwon B., Lee M., Waring A. J. & Hong M. Oligomeric Structure and Three-Dimensional Fold of the HIV gp41 Membrane-Proximal External Region and Transmembrane Domain in Phospholipid Bilayers. J. Am. Chem. Soc. 140, 8246–8259 (2018). 29888593 10.1021/jacs.8b04010 PMC6382510

[12] Piai A. Structural basis of transmembrane coupling of the HIV-1 envelope glycoprotein. Nat. Commun. 11, 2317 (2020). 32385256 10.1038/s41467-020-16165-0 PMC7210310

[13] Piai A. NMR Model of the Entire Membrane-Interacting Region of the HIV-1 Fusion Protein and Its Perturbation of Membrane Morphology. J. Am. Chem. Soc. 143, 6609–6615 (2021). 33882664 10.1021/jacs.1c01762

[14] Apellaniz B. The Atomic Structure of the HIV-1 gp41 Transmembrane Domain and Its Connection to the Immunogenic Membrane-proximal External Region. J. Biol. Chem. 290, 12999–13015 (2015). 25787074 10.1074/jbc.M115.644351 PMC4505554

[15] Reardon P. N. Structure of an HIV-1-neutralizing antibody target, the lipid-bound gp41 envelope membrane proximal region trimer. Proc. Natl. Acad. Sci. U.S.A. 111, 1391–1396 (2014). 24474763 10.1073/pnas.1309842111 PMC3910588

[16] Sun Z. Y. HIV-1 broadly neutralizing antibody extracts its epitope from a kinked gp41 ectodomain region on the viral membrane. Immunity 28, 52–63 (2008). 18191596 10.1016/j.immuni.2007.11.018

[17] Chiliveri S. C., Louis J. M., Ghirlando R., Baber J. L. & Bax A. Tilted, Uninterrupted, Monomeric HIV-1 gp41 Transmembrane Helix from Residual Dipolar Couplings. J. Am. Chem. Soc. 140, 34–37 (2018). 29277995 10.1021/jacs.7b10245 PMC6121722

[18] Checkley M. A., Luttge B. G. & Freed E. O. HIV-1 envelope glycoprotein biosynthesis, trafficking, and incorporation. J. Mol. Biol. 410, 582–608 (2011). 21762802 10.1016/j.jmb.2011.04.042 PMC3139147

[19] Murphy R. E., Samal A. B., Vlach J. & Saad J. S. Solution Structure and Membrane Interaction of the Cytoplasmic Tail of HIV-1 gp41 Protein. Structure 25, 1708–1718 e1705 (2017). 29056482 10.1016/j.str.2017.09.010 PMC5687296

[20] Pogozheva I. D. Comparative Molecular Dynamics Simulation Studies of Realistic Eukaryotic, Prokaryotic, and Archaeal Membranes. J. Chem. Inf. Model. 62, 1036–1051 (2022). 35167752 10.1021/acs.jcim.1c01514

[21] Humphrey W., Dalke A. & Schulten K. VMD: visual molecular dynamics. J. Mol. Graph. 14, 33–38, 27–38 (1996). 8744570 10.1016/0263-7855(96)00018-5

[22] Cheng X. & Im W. NMR observable-based structure refinement of DAP12-NKG2C activating immunoreceptor complex in explicit membranes. Biophys. J. 102, L27–29 (2012). 22500771 10.1016/j.bpj.2012.03.002 PMC3318141

[23] Fu Q. Structure of the membrane proximal external region of HIV-1 envelope glycoprotein. Proc. Natl. Acad. Sci. U.S.A. 115, E8892–E8899 (2018). 30185554 10.1073/pnas.1807259115 PMC6156635

[24] Williams L. D. Potent and broad HIV-neutralizing antibodies in memory B cells and plasma. Sci. Immunol. 2 (2017). 10.1126/sciimmunol.aal2200 PMC5905719 28783671

[25] Behrens A. J. Composition and Antigenic Effects of Individual Glycan Sites of a Trimeric HIV-1 Envelope Glycoprotein. Cell Rep. 14, 2695–2706 (2016). 26972002 10.1016/j.celrep.2016.02.058 PMC4805854

[26] Cao L. Global site-specific N-glycosylation analysis of HIV envelope glycoprotein. Nat. Commun. 8, 14954 (2017). 28348411 10.1038/ncomms14954 PMC5379070

[27] Chen J. Mechanism of HIV-1 neutralization by antibodies targeting a membrane-proximal region of gp41. J. Virol. 88, 1249–1258 (2014). 24227838 10.1128/JVI.02664-13 PMC3911647

[28] Alam S. M. Role of HIV membrane in neutralization by two broadly neutralizing antibodies. Proc. Natl. Acad. Sci. U.S.A. 106, 20234–20239 (2009). 19906992 10.1073/pnas.0908713106 PMC2787149

[29] Lee J. H., Ozorowski G. & Ward A. B. Cryo-EM structure of a native, fully glycosylated, cleaved HIV-1 envelope trimer. Science 351, 1043–1048 (2016). 26941313 10.1126/science.aad2450 PMC5001164

[30] Sarkar A. Structure of a cleavage-independent HIV Env recapitulates the glycoprotein architecture of the native cleaved trimer. Nat. Commun. 9, 1956 (2018). 29769533 10.1038/s41467-018-04272-y PMC5955915

[31] Yang J. & Zhang Y. I-TASSER server: new development for protein structure and function predictions. Nucleic Acids Res. 43, W174–181 (2015). 25883148 10.1093/nar/gkv342 PMC4489253

[32] Jo S., Song K. C., Desaire H., MacKerell A. D. Jr. & Im W. Glycan Reader: automated sugar identification and simulation preparation for carbohydrates and glycoproteins. J. Comput. Chem. 32, 3135–3141 (2011). 21815173 10.1002/jcc.21886 PMC3188666

[33] Park S. J. Glycan Reader is improved to recognize most sugar types and chemical modifications in the Protein Data Bank. Bioinformatics 33, 3051–3057 (2017). 28582506 10.1093/bioinformatics/btx358 PMC5870669

[34] Park S. J. CHARMM-GUI Glycan Modeler for modeling and simulation of carbohydrates and glycoconjugates. Glycobiology 29, 320–331 (2019). 30689864 10.1093/glycob/cwz003 PMC6422236

[35] Jo S., Kim T., Iyer V. G. & Im W. CHARMM-GUI: a web-based graphical user interface for CHARMM. J. Comput. Chem. 29, 1859–1865 (2008). 18351591 10.1002/jcc.20945

[36] Jorgensen W. L., Chandrasekhar J., Madura J. D., Impey R. W. & Klein M. L. Comparison of simple potential functions for simulating liquid water. J. Chem. Phys. 79, 926–935 (1983).

[37] Jo S., Kim T. & Im W. Automated builder and database of protein/membrane complexes for molecular dynamics simulations. PLoS One 2, e880 (2007). 17849009 10.1371/journal.pone.0000880 PMC1963319

[38] Jo S., Lim J. B., Klauda J. B. & Im W. CHARMM-GUI Membrane Builder for mixed bilayers and its application to yeast membranes. Biophys. J. 97, 50–58 (2009). 19580743 10.1016/j.bpj.2009.04.013 PMC2711372

[39] Wu E. L. CHARMM-GUI Membrane Builder toward realistic biological membrane simulations. J. Comput. Chem. 35, 1997–2004 (2014). 25130509 10.1002/jcc.23702 PMC4165794

[40] Lee J. CHARMM-GUI Input Generator for NAMD, GROMACS, AMBER, OpenMM, and CHARMM/OpenMM Simulations Using the CHARMM36 Additive Force Field. J. Chem. Theory. Comput. 12, 405–413 (2016). 26631602 10.1021/acs.jctc.5b00935 PMC4712441

[41] Lee J. CHARMM-GUI Membrane Builder for Complex Biological Membrane Simulations with Glycolipids and Lipoglycans. J. Chem. Theory. Comput. 15, 775–786 (2019). 30525595 10.1021/acs.jctc.8b01066

[42] Huang J. CHARMM36m: an improved force field for folded and intrinsically disordered proteins. Nat. Methods 14, 71–73 (2017). 27819658 10.1038/nmeth.4067 PMC5199616

[43] Guvench O., Hatcher E. R., Venable R. M., Pastor R. W. & Mackerell A. D. CHARMM Additive All-Atom Force Field for Glycosidic Linkages between Hexopyranoses. J. Chem. Theory. Comput. 5, 2353–2370 (2009). 20161005 10.1021/ct900242e PMC2757763

[44] Raman E. P., Guvench O. & MacKerell A. D. Jr. CHARMM additive all-atom force field for glycosidic linkages in carbohydrates involving furanoses. J. Phys. Chem. B 114, 12981–12994 (2010). 20845956 10.1021/jp105758h PMC2958709

[45] Guvench O. CHARMM additive all-atom force field for carbohydrate derivatives and its utility in polysaccharide and carbohydrate-protein modeling. J. Chem. Theory. Comput. 7, 3162–3180 (2011). 22125473 10.1021/ct200328p PMC3224046

[46] Klauda J. B. Update of the CHARMM all-atom additive force field for lipids: validation on six lipid types. J. Phys. Chem. B 114, 7830–7843 (2010). 20496934 10.1021/jp101759q PMC2922408

[47] Steinbach P. J. & Brooks B. R. New spherical-cutoff methods for long-range forces in macromolecular simulation. J. Comput. Chem. 15, 667–683 (2004).

[48] Essmann U. A smooth particle mesh Ewald method. J. Chem. Phys. 103, 8577–8593 (1995).

[49] Van Der Spoel D. GROMACS: fast, flexible, and free. J. Comput. Chem. 26, 1701–1718 (2005). 16211538 10.1002/jcc.20291

[50] Hess B., Bekker H., Berendsen H. J. C. & Fraaije J. G. E. M. LINCS: A linear constraint solver for molecular simulations. J. Comput. Chem. 18, 1463–1472 (1997).

[51] Berendsen H. J. C., Postma J. P. M., van Gunsteren W. F., DiNola A. & Haak J. R. Molecular dynamics with coupling to an external bath. J. Chem. Phys. 81, 3684–3690 (1984).

[52] Nosé S. A molecular dynamics method for simulations in the canonical ensemble. Mol. Phys. 52, 255–268 (2006).

[53] Hoover W. G. Canonical dynamics: Equilibrium phase-space distributions. Phys. Rev. A Gen. Phys. 31, 1695–1697 (1985). 9895674 10.1103/physreva.31.1695

[54] Parrinello M. & Rahman A. Polymorphic transitions in single crystals: A new molecular dynamics method. J. Appl. Phys. 52, 7182–7190 (1981).

[55] Nosé S. & Klein M. L. Constant pressure molecular dynamics for molecular systems. Mol. Phys. 50, 1055–1076 (2006).

[56] Hopkins C. W., Le Grand S., Walker R. C. & Roitberg A. E. Long-Time-Step Molecular Dynamics through Hydrogen Mass Repartitioning. J. Chem. Theory. Comput. 11, 1864–1874 (2015). 26574392 10.1021/ct5010406

[57] McGibbon R. T. MDTraj: A Modern Open Library for the Analysis of Molecular Dynamics Trajectories. Biophys. J. 109, 1528–1532 (2015). 26488642 10.1016/j.bpj.2015.08.015 PMC4623899

[58] McLellan J. S. Structure of HIV-1 gp120 V1/V2 domain with broadly neutralizing antibody PG9. Nature 480, 336–343 (2011). 22113616 10.1038/nature10696 PMC3406929

[59] Zhou T. Multidonor analysis reveals structural elements, genetic determinants, and maturation pathway for HIV-1 neutralization by VRC01-class antibodies. Immunity 39, 245–258 (2013). 23911655 10.1016/j.immuni.2013.04.012 PMC3985390

[60] Pancera M. Structure and immune recognition of trimeric pre-fusion HIV-1 Env. Nature 514, 455–461 (2014). 25296255 10.1038/nature13808 PMC4348022

[61] Rantalainen K. HIV-1 Envelope and MPER Antibody Structures in Lipid Assemblies. Cell Rep. 31, 107583 (2020). 32348769 10.1016/j.celrep.2020.107583 PMC7196886

[62] Zhang Y. & Skolnick J. TM-align: a protein structure alignment algorithm based on the TM-score. Nucleic Acids Res. 33, 2302–2309 (2005). 15849316 10.1093/nar/gki524 PMC1084323

