# Supplementary material for: Conformational Variability of HIV-1 Env Trimer and Viral Vulnerability
Source: bioRxiv. 2025 Oct 10:2025.10.09.681376. Preprint. [Version 1] doi: 10.1101/2025.10.09.681376 (PMC12632500; doi:10.1101/2025.10.09.681376)
Supplement: Supplement 1 [file media-1.pdf]

Supporting Information

Conformational Variability of HIV-1 Env Trimer and Viral Vulnerability

Yiwei Cao[1] and Wonpil Im[1*]

[1]Department of Biological Sciences, Lehigh University, 111 Research Dr, Bethlehem, PA 18015, USA

[*]Corresponding Author: Wonpil Im (wonpil@lehigh.edu)

Table S1. Simulation system information.

Name	Cleaved	TMD Position	CT Truncated	Number of Atoms	Simulation Time	Number of Simulation Runs
$CH^{\Delta CT}$	Yes	High	Yes	1,046,227	1 μs	3
$CL^{\Delta CT}$	Yes	Low	Yes	1,040,888	1 μs	3
$UH^{\Delta CT}$	No	High	Yes	1,080,915	1 μs	3
$UL^{\Delta CT}$	No	Low	Yes	1,080,164	1 μs	3
CH^{CT}	Yes	High	No	1,080,113	1 μs	3
CL^{CT}	Yes	Low	No	1,078,556	1 μs	3
UH^{CT}	No	High	No	1,118,568	1 μs	3
UL^{CT}	No	Low	No	1,118,375	1 μs	3

Table S2. Frequency of epitope accessibility for antibody PGT128.

	$CH^{\Delta CT}1$	$CH^{\Delta CT}2$	$CH^{\Delta CT}3$	$CL^{\Delta CT}1$	$CL^{\Delta CT}2$	$CL^{\Delta CT}3$
A[1]	19%	7%[4]	36%	21%	16%	1%
B[1]	76%	1%	15%	19%	3%	46%
C[1]	43%	13%	17%	19%	14%	19%
A&B[2]	17%	0%	8%	8%	1%	0%
A&C[2]	6%	0%	1%	6%	0%	0%
B&C[2]	37%	0%	3%	2%	0%	14%
A&B&C[3]	6%	0%	0%	1%	0%	0%

	$UH^{\Delta CT}1$	$UH^{\Delta CT}2$	$UH^{\Delta CT}3$	$UL^{\Delta CT}1$	$UL^{\Delta CT}2$	$UL^{\Delta CT}3$
A	5%	32%	2%	3%	9%	5%
B	35%	38%	41%	2%	28%	2%
C	12%	16%	53%	45%	3%	33%
A&B	1%	15%	1%	0%	5%	0%
A&C	1%	9%	1%	1%	1%	2%
B&C	4%	10%	20%	1%	2%	1%
A&B&C	1%	5%	1%	0%	1%	0%

	$CH^{CT}1$	$CH^{CT}2$	$CH^{CT}3$	$CL^{CT}1$	$CL^{CT}2$	$CL^{CT}3$
A	7%	10%	9%	15%	0%	7%
B	2%	15%	19%	22%	0%	0%
C	44%	2%	25%	14%	4%	23%
A&B	0%	2%	0%	3%	0%	0%
A&C	1%	0%	1%	1%	0%	1%
B&C	1%	0%	9%	5%	0%	0%
A&B&C	0%	0%	0%	1%	0%	0%

	$UH^{CT}1$	$UH^{CT}2$	$UH^{CT}3$	$UL^{CT}1$	$UL^{CT}2$	$UL^{CT}3$
A	1%	1%	26%	33%	1%	2%
B	2%	23%	26%	45%	67%	48%
C	4%	34%	13%	3%	38%	17%
A&B	0%	1%	5%	14%	1%	0%
A&C	0%	1%	1%	2%	1%	1%
B&C	0%	9%	3%	2%	29%	1%
A&B&C	0%	1%	0%	1%	1%	0%

[1]Three protein chains are referred to as A, B, and C. [2]"A&B" denotes that the epitopes on both chains A and B are concurrently accessible. [3]"A&B&C" denotes that the epitopes on all three chains are concurrently accessible. [4]Red text highlights cases where the frequency of epitope accessibility on an individual chain is <10%. The same notations apply for **Tables S3 and S4**.

Table S3. Frequency of epitope accessibility for antibody PG9.

	$CH^{\Delta CT}1$	$CH^{\Delta CT}2$	$CH^{\Delta CT}3$	$CL^{\Delta CT}1$	$CL^{\Delta CT}2$	$CL^{\Delta CT}3$
A	0%	0%	0%	0%	0%	0%
B	20%	34%	1%	11%	0%	15%
C	0%	0%	1%	0%	0%	0%
A&B	0%	0%	0%	0%	0%	0%
A&C	0%	0%	0%	0%	0%	0%
B&C	0%	0%	0%	0%	0%	0%
A&B&C	0%	0%	0%	0%	0%	0%

	$UH^{\Delta CT}1$	$UH^{\Delta CT}2$	$UH^{\Delta CT}3$	$UL^{\Delta CT}1$	$UL^{\Delta CT}2$	$UL^{\Delta CT}3$
A	1%	0%	0%	0%	1%	0%
B	0%	1%	2%	0%	1%	0%
C	2%	0%	0%	0%	0%	0%
A&B	0%	0%	0%	0%	0%	0%
A&C	0%	0%	0%	0%	0%	0%
B&C	0%	0%	0%	0%	0%	0%
A&B&C	0%	0%	0%	0%	0%	0%

	$CH^{CT}1$	$CH^{CT}2$	$CH^{CT}3$	$CL^{CT}1$	$CL^{CT}2$	$CL^{CT}3$
A	0%	0%	0%	0%	0%	0%
B	1%	0%	2%	3%	0%	31%
C	0%	0%	0%	0%	5%	0%
A&B	0%	0%	0%	0%	0%	0%
A&C	0%	0%	0%	0%	0%	0%
B&C	0%	0%	0%	0%	0%	0%
A&B&C	0%	0%	0%	0%	0%	0%

	$UH^{CT}1$	$UH^{CT}2$	$UH^{CT}3$	$UL^{CT}1$	$UL^{CT}2$	$UL^{CT}3$
A	0%	1%	1%	0%	0%	0%
B	0%	1%	0%	0%	0%	0%
C	0%	1%	0%	0%	2%	0%
A&B	0%	0%	0%	0%	0%	0%
A&C	0%	0%	0%	0%	0%	0%
B&C	0%	0%	0%	0%	0%	0%
A&B&C	0%	0%	0%	0%	0%	0%

Table S4. Frequency of epitope accessibility for antibody VRC01.

	$CH^{\Delta CT}1$	$CH^{\Delta CT}2$	$CH^{\Delta CT}3$	$CL^{\Delta CT}1$	$CL^{\Delta CT}2$	$CL^{\Delta CT}3$
A	0%	0%	10%	18%	0%	4%
B	2%	2%	5%	4%	20%	5%
C	41%	3%	1%	0%	3%	4%
A&B	0%	0%	0%	0%	0%	0%
A&C	0%	0%	0%	0%	0%	0%
B&C	0%	0%	0%	0%	1%	0%
A&B&C	0%	0%	0%	0%	0%	0%

	$UH^{\Delta CT}1$	$UH^{\Delta CT}2$	$UH^{\Delta CT}3$	$UL^{\Delta CT}1$	$UL^{\Delta CT}2$	$UL^{\Delta CT}3$
A	15%	0%	1%	7%	15%	4%
B	4%	0%	1%	0%	7%	13%
C	24%	5%	32%	33%	6%	45%
A&B	0%	0%	0%	0%	2%	0%
A&C	7%	0%	1%	1%	1%	1%
B&C	0%	0%	0%	0%	0%	10%
A&B&C	0%	0%	0%	0%	0%	0%

	$CH^{CT}1$	$CH^{CT}2$	$CH^{CT}3$	$CL^{CT}1$	$CL^{CT}2$	$CL^{CT}3$
A	14%	13%	0%	0%	2%	9%
B	9%	3%	1%	4%	2%	3%
C	9%	11%	10%	0%	0%	24%
A&B	2%	0%	0%	0%	0%	2%
A&C	1%	2%	0%	0%	0%	3%
B&C	1%	0%	0%	0%	0%	0%
A&B&C	0%	0%	0%	0%	0%	0%

	$UH^{CT}1$	$UH^{CT}2$	$UH^{CT}3$	$UL^{CT}1$	$UL^{CT}2$	$UL^{CT}3$
A	17%	0%	16%	4%	22%	5%
B	6%	0%	1%	0%	0%	0%
C	57%	19%	57%	35%	14%	44%
A&B	2%	0%	0%	0%	0%	0%
A&C	6%	0%	11%	2%	2%	0%
B&C	2%	0%	1%	0%	0%	0%
A&B&C	0%	0%	0%	0%	0%	0%

Table S5. Frequency of epitope accessibility for antibody 35O22.

	$CH^{\Delta CT}1$	$CH^{\Delta CT}2$	$CH^{\Delta CT}3$	$CL^{\Delta CT}1$	$CL^{\Delta CT}2$	$CL^{\Delta CT}3$
A	7%, 0%[1]	4%, 0%	9%, 8%	3%, 0%	24%, 0%	7%, 4%
B	29%, 2%[2]	14%, 0%	12%, 0%	0%, 0%	10%, 10%	0%, 0%
C	8%, 8%	14%, 10%	10%, 5%	32%, 7%	4%, 1%	22%, 16%
A&B	7%, 0%	4%, 0%	9%, 0%	0%, 0%	10%, 0%	0%, 0%
A&C	7%, 0%	4%, 0%	9%, 5%	3%, 0%	4%, 0%	7%, 4%
B&C	8%, 2%	14%, 0%	10%, 0%	0%, 0%	4%, 1%	0%, 0%
A&B&C	7%, 0%	4%, 0%	9%, 0%	0%, 0%	4%, 0%	0%, 0%

	$UH^{\Delta CT}1$	$UH^{\Delta CT}2$	$UH^{\Delta CT}3$	$UL^{\Delta CT}1$	$UL^{\Delta CT}2$	$UL^{\Delta CT}3$
A	16%, 12%	49%, 1%	9%, 7%	14%, 5%	23%, 19%	43%, 10%
B	2%, 2%	5%, 5%	10%, 9%	7%, 7%	3%, 1%	5%, 0%
C	22%, 13%	25%, 25%	23%, 23%	26%, 3%	6%, 5%	5%, 5%
A&B	2%, 2%	5%, 1%	9%, 7%	7%, 5%	3%, 1%	5%, 0%
A&C	16%, 12%	25%, 1%	9%, 7%	14%, 3%	6%, 5%	5%, 5%
B&C	2%, 2%	5%, 5%	10%, 9%	7%, 3%	3%, 1%	5%, 0%
A&B&C	2%, 2%	5%, 1%	9%, 7%	7%, 3%	3%, 1%	5%, 0%

	$CH^{CT}1$	$CH^{CT}2$	$CH^{CT}3$	$CL^{CT}1$	$CL^{CT}2$	$CL^{CT}3$
A	9%, 0%	30%, 0%	48%, 18%	12%, 4%	22%, 0%	5%, 5%
B	0%, 0%	0%, 0%	31%, 8%	11%, 11%	24%, 11%	24%, 1%
C	2%, 2%	3%, 2%	7%, 0%	24%, 0%	6%, 2%	2%, 2%
A&B	0%, 0%	0%, 0%	31%, 8%	11%, 4%	22%, 0%	5%, 1%
A&C	2%, 0%	3%, 0%	7%, 0%	12%, 0%	6%, 0%	2%, 2%
B&C	0%, 0%	0%, 0%	7%, 0%	11%, 0%	6%, 2%	2%, 1%
A&B&C	0%, 0%	0%, 0%	7%, 0%	11%, 0%	6%, 0%	2%, 1%

	$UH^{CT}1$	$UH^{CT}2$	$UH^{CT}3$	$UL^{CT}1$	$UL^{CT}2$	$UL^{CT}3$
A	5%, 4%	18%, 16%	33%, 0%	31%, 0%	31%, 0%	41%, 0%
B	26%, 16%	8%, 5%	11%, 7%	2%, 1%	2%, 0%	18%, 1%
C	25%, 0%	7%, 0%	20%, 9%	3%, 2%	29%, 15%	7%, 7%
A&B	5%, 4%	8%, 5%	11%, 0%	2%, 0%	2%, 0%	18%, 0%
A&C	5%, 0%	7%, 0%	20%, 0%	3%, 0%	29%, 0%	7%, 0%
B&C	25%, 0%	7%, 0%	11%, 7%	2%, 1%	2%, 0%	7%, 1%
A&B&C	5%, 0%	7%, 0%	11%, 0%	2%, 0%	2%, 0%	7%, 0%

[1]The first value represents the frequency of epitope accessibility considering only glycan shielding, and the second value represents the frequency considering the shielding of both glycan and membrane. Red text highlights cases where the first value is <10%. [2]Magenta text highlights cases where the first value is ≥10% but the second value is <10%.

Table S6. Glycosylation sites, selected glycan compositions, and sequences used in this study.

Glycosylation Site	Composition and Type	Sequence
N197, N355[1]	HexNAc(2)Hex(5) High-mannose	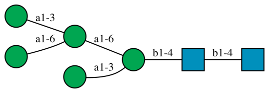
N276[1]	HexNAc(2)Hex(7) High-mannose	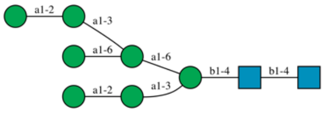
N160[1]	HexNAc(2)Hex(8) High-mannose	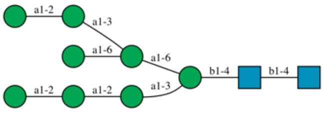
N133, N156, N234, N262, N295, N301, N332, N339, N363, N386, N392, N411, N448	HexNAc(2)Hex(9) High-mannose	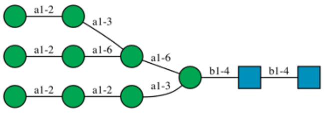
N88, N137, N185E, N185H, N398, N406, N618, N637[2]	HexNAc(4)Hex(5) Fuc(1)Neu5Ac(2) Complex	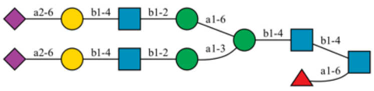
N462, N611[2,3]	HexNAc(5)Hex(6) Fuc(1)Neu5Ac(3) Complex	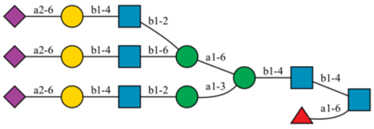

[1]The same composition can correspond to multiple possible sequences, depending on which branch the mannose (Man) is attached to. In such cases, one representative sequence was selected for structural modeling. [2]N-Acetylneuraminic acid (Neu5Ac) can be either α2-3 or α2-6 linked to galactose (Gal). In this study, the α2-6 linkage was used for all non-reducing terminal Neu5Ac. [3]Both the α1-3 and α1-6 branches can contain one or two antennae. Here, we added one antenna to the α1-3 branch and two antennae to the α1-6 branch.

Table S7. Membrane lipid composition.

CT-truncated (ΔCT)

Lipid Name	Lipid Head/Tail	Number of Lipids in Leaflets	
		Exoplasmic	Cytoplasmic
POPC	PC (16:0/18:1(9Z))	120	56
PLPC	PC (16:0/18:2(9Z,12Z))	165	88
PAPE	PE (16:0/20:4(5Z,8Z,11Z,14Z))	23	96
POPE	PE (16:0/18:1(9Z))	23	112
POPI	PI (16:0/18:1(9Z))	0	40
PAPS	PS (16:0/20:4(5Z,8Z,11Z,14Z))	0	88
POPA	PA (16:0/18:1(9Z))	0	8
SSM	SM (d18:1/18:0)	83	40
NSM	SM (d18:1/24:1)	83	40
CMH	GlcCer (d18:1/16:0)	30	0
CHOL	Cholesterol	278	232
TOTAL		805	800

Full-length (CT)

Lipid Name	Lipid Head/Tail	Number of Lipids in Leaflets	
		Exoplasmic	Cytoplasmic
POPC	PC (16:0/18:1(9Z))	120	49
PLPC	PC (16:0/18:2(9Z,12Z))	165	77
PAPE	PE (16:0/20:4(5Z,8Z,11Z,14Z))	23	84
POPE	PE (16:0/18:1(9Z))	23	98
POPI	PI (16:0/18:1(9Z))	0	35
PAPS	PS (16:0/20:4(5Z,8Z,11Z,14Z))	0	77
POPA	PA (16:0/18:1(9Z))	0	7
SSM	SM (d18:1/18:0)	83	35
NSM	SM (d18:1/24:1)	83	35
CMH	GlcCer (d18:1/16:0)	30	0
CHOL	Cholesterol	278	203
TOTAL		805	700

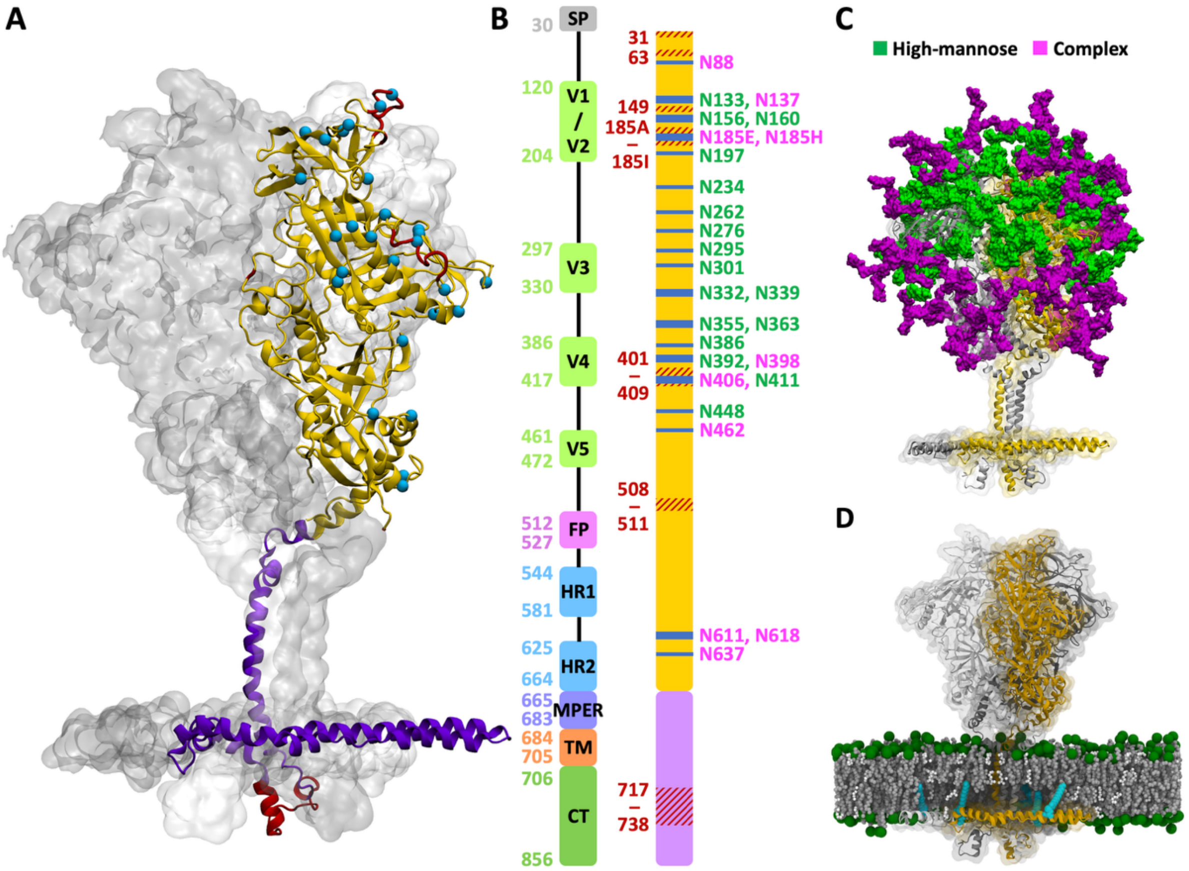

Figure S1. Model structure of the fully glycosylated full-length HIV Env trimer embedded in a membrane, including the cytoplasmic tail. Labeling and color coding are the same as in **Figure 1**. (D) The palmitoyl groups covalently attached to C764 and C837 are shown in cyan.

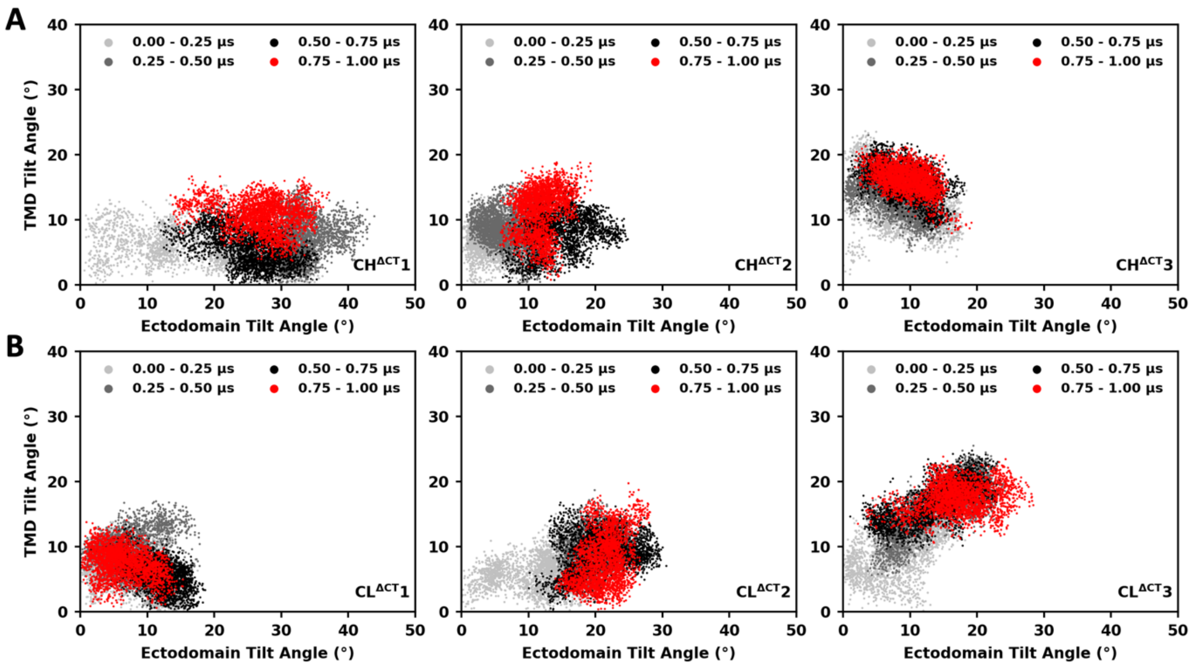

Figure S2. Ectodomain tilt versus TMD tilt, grouped by time intervals (cleaved CT-truncated systems). (A) Three trajectories starting from the "high" TMD configuration. (B) Three trajectories starting from the "low" TMD configuration. The 1-µs trajectory was divided into four intervals, with values from each interval shown in light gray, dark gray, black, and red, respectively.

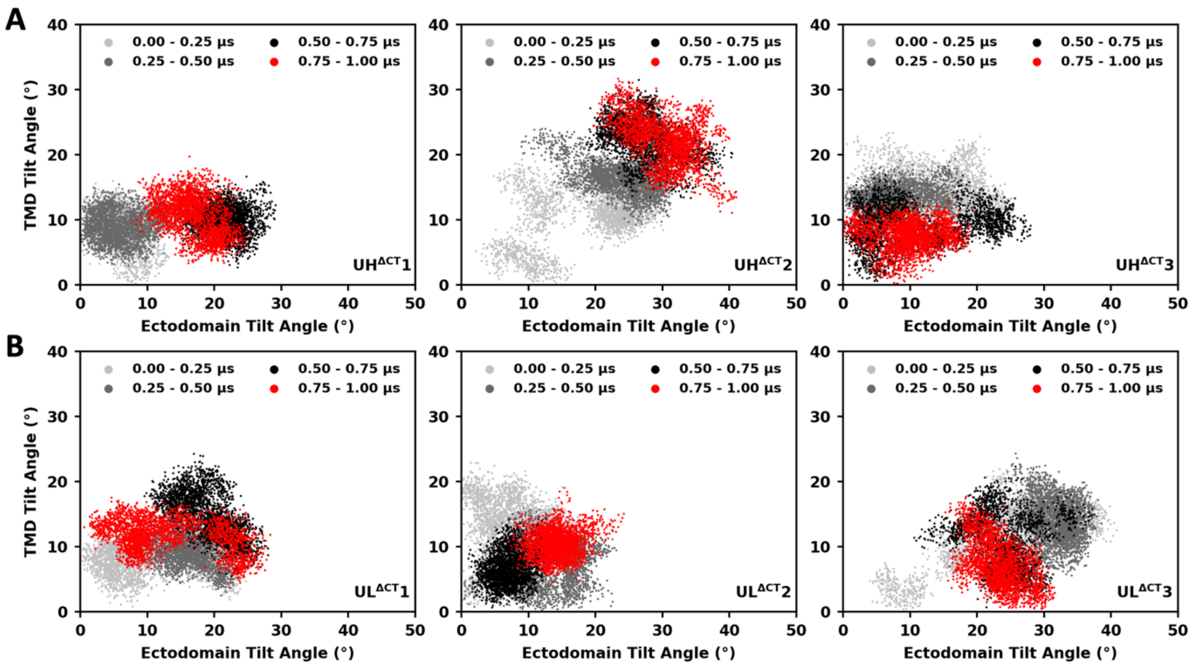

Figure S3. Ectodomain tilt versus TMD tilt, grouped by time intervals (uncleaved CT-truncated systems). Labeling and color coding are the same as in **Figure S2**.

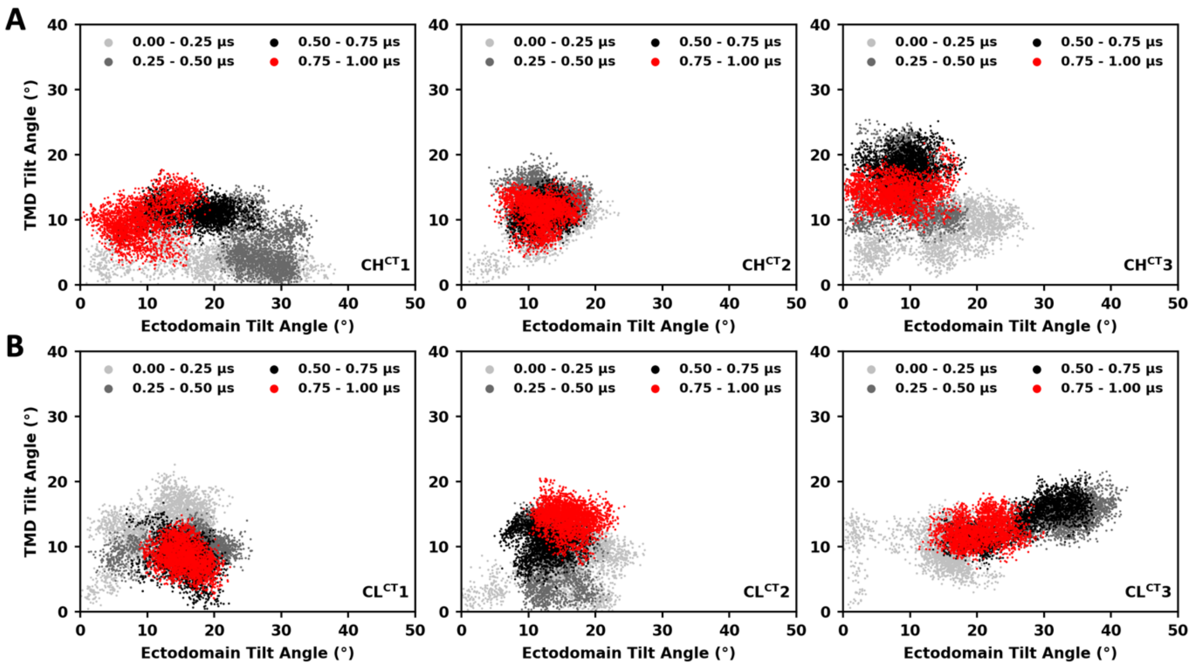

Figure S4. Ectodomain tilt versus TMD tilt, grouped by time intervals (cleaved full-length systems). Labeling and color coding are the same as in **Figure S2**.

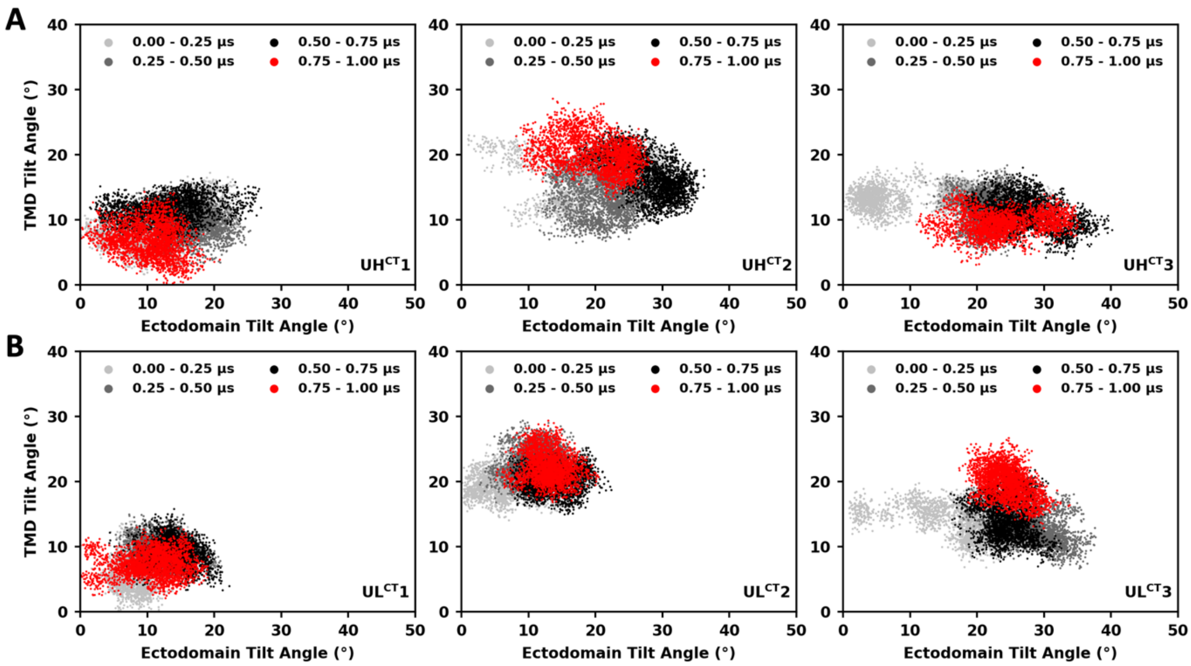

Figure S5. Ectodomain tilt versus TMD tilt, grouped by time intervals (uncleaved full-length systems). Labeling and color coding are the same as in **Figure S2**.

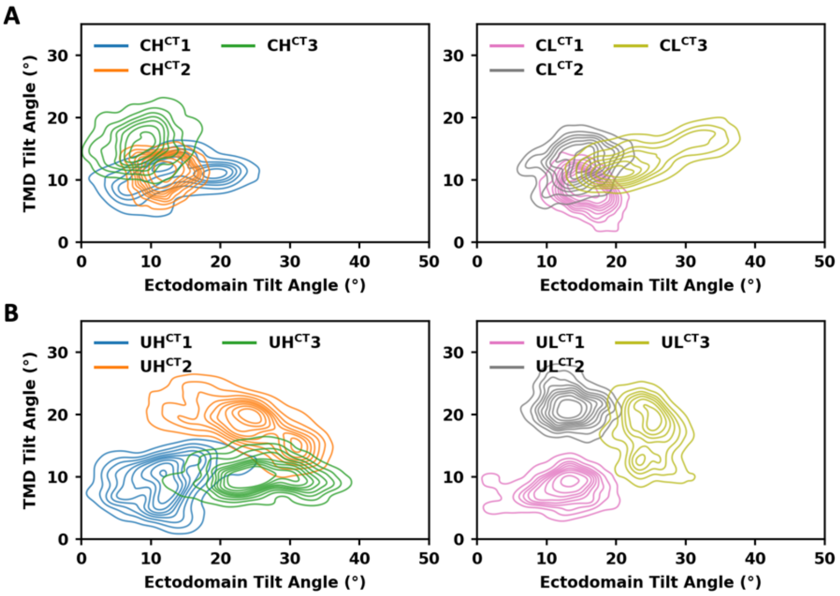

Figure S6. Ectodomain tilt versus TMD tilt (full-length systems).

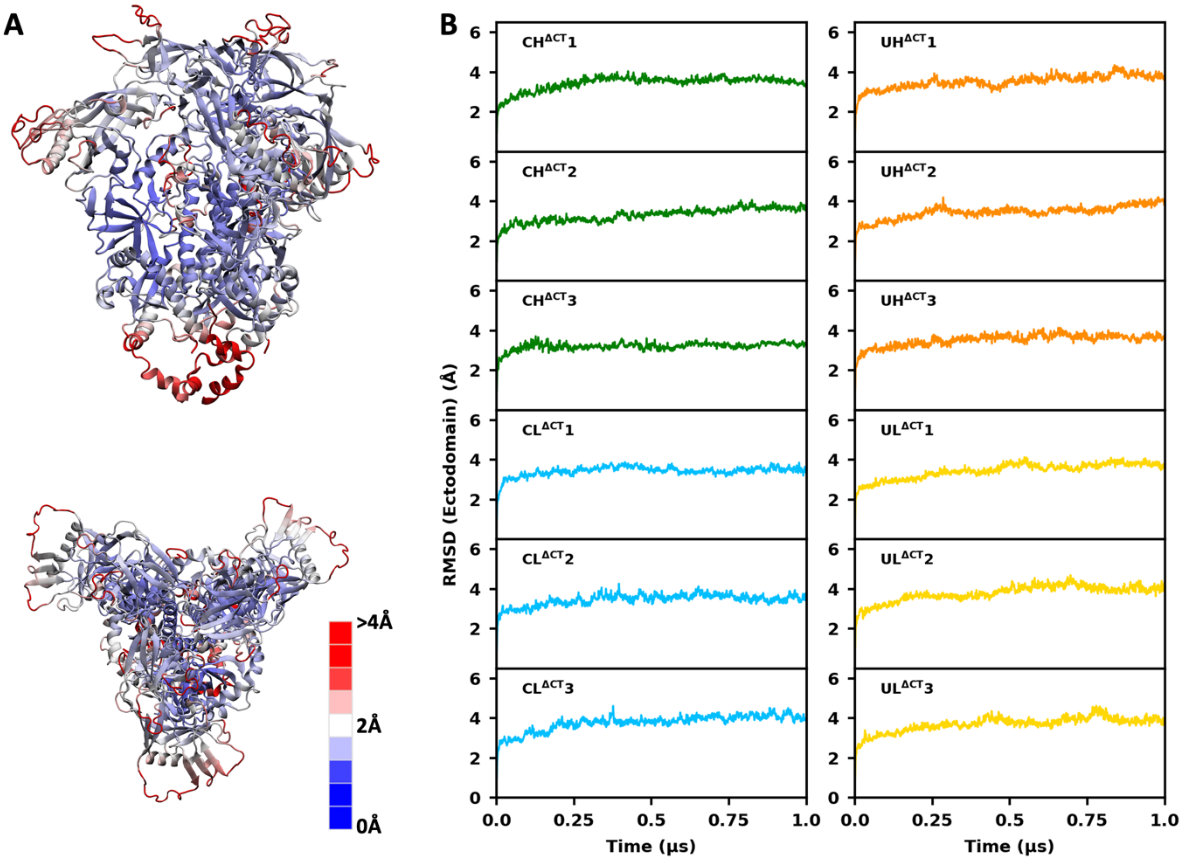

Figure S7. RMSF and RMSD of the ectodomain. (A) Top and side views of the ectodomain and MPER in the uncleaved system, with RMSF indicated by color. (B) RMSD relative to the initial model as a function of time, calculated from the trajectories of CT-truncated systems.

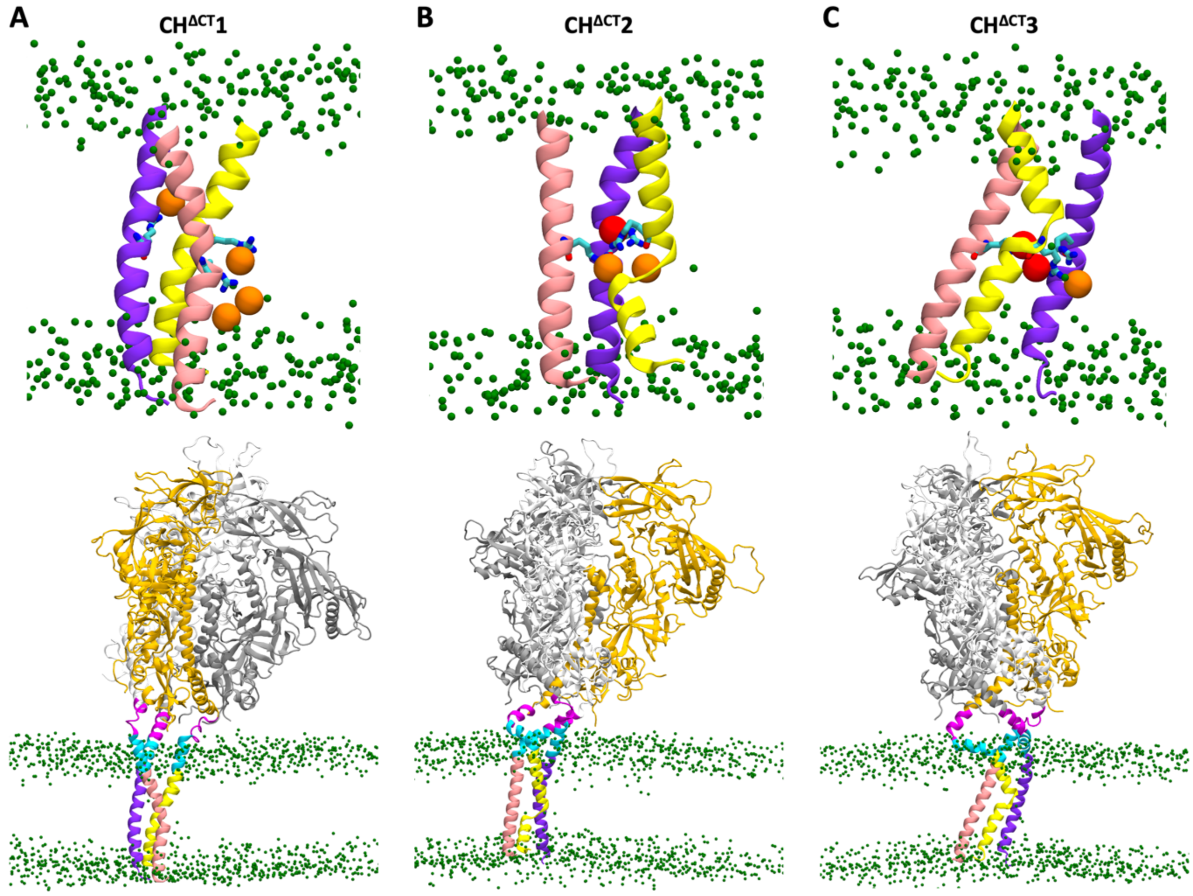

Figure S8. Local conformations of the TMD and global conformations of protein and membrane ($CH^{ΔCT}$ systems). Three protomers of the TMD are shown in light yellow, purple, and pink; three protomers of the ectodomain in dark yellow, gray, and white; MPER-N and MPER-C in magenta and cyan, respectively; and lipid headgroups in green. Lipid headgroups interacting with R696 are highlighted in orange, and the ions interacting with R696 in red.

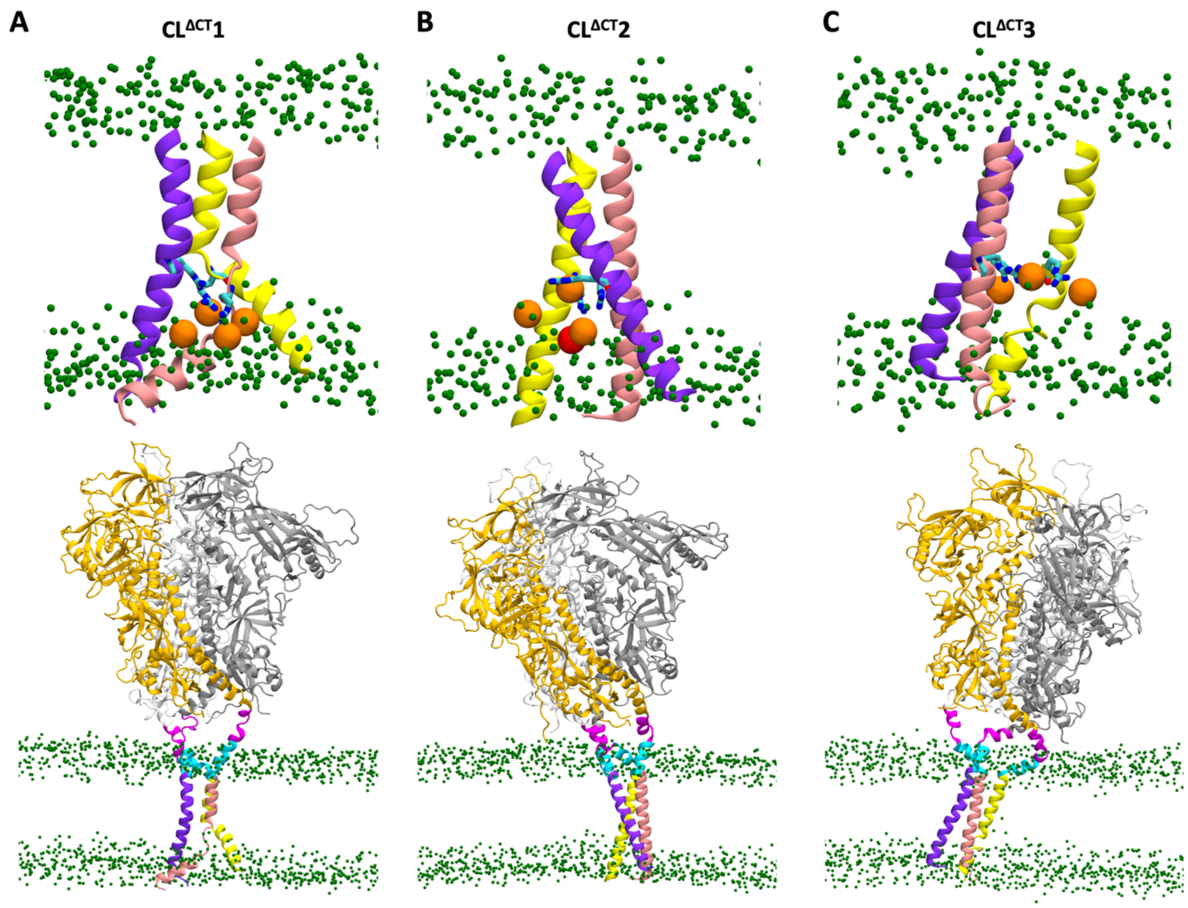

Figure S9. Local conformations of the TMD and global conformations of protein and membrane (CL$^{\Delta CT}$ systems). Labeling and color coding are the same as in **Figure S8**.

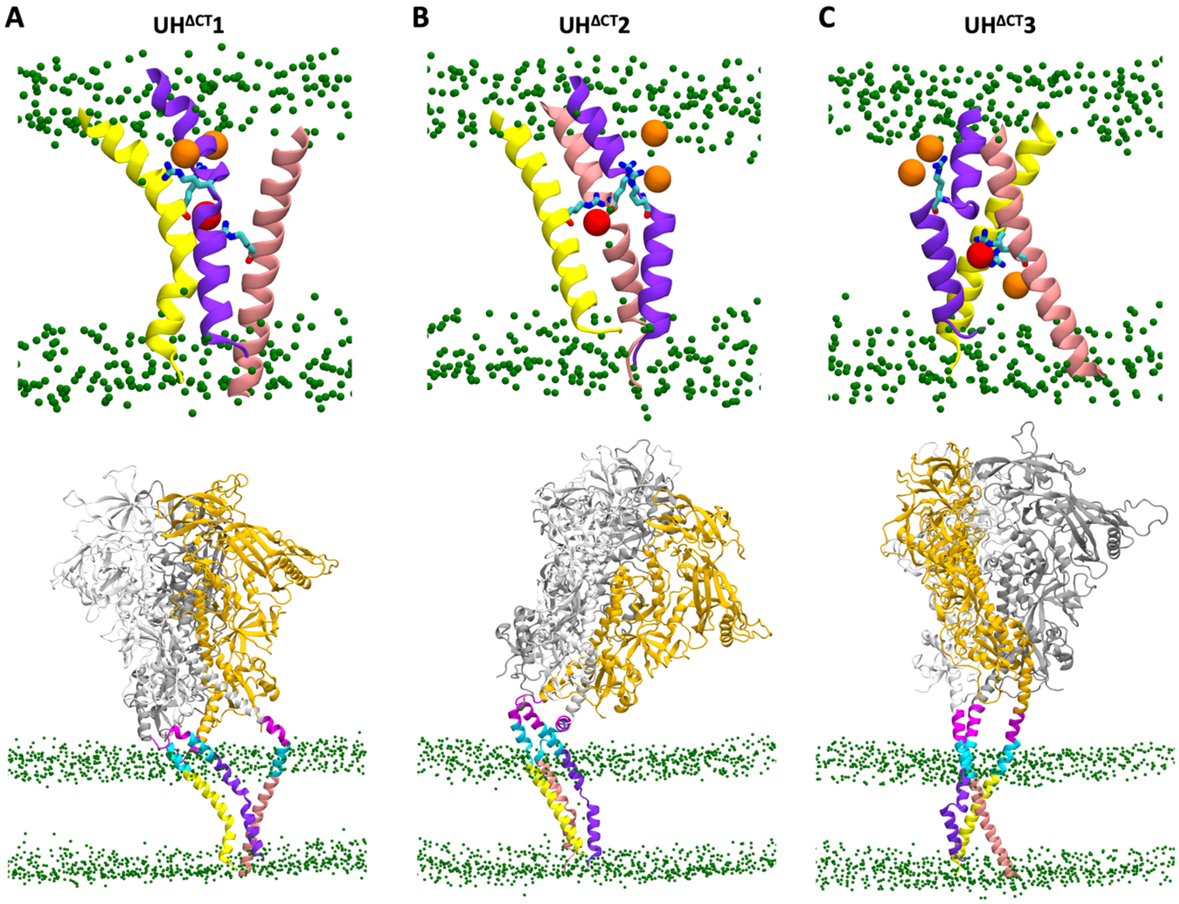

Figure S10. Local conformations of the TMD and global conformations of protein and membrane (UH$^{\Delta CT}$ systems). Labeling and color coding are the same as in **Figure S8**.

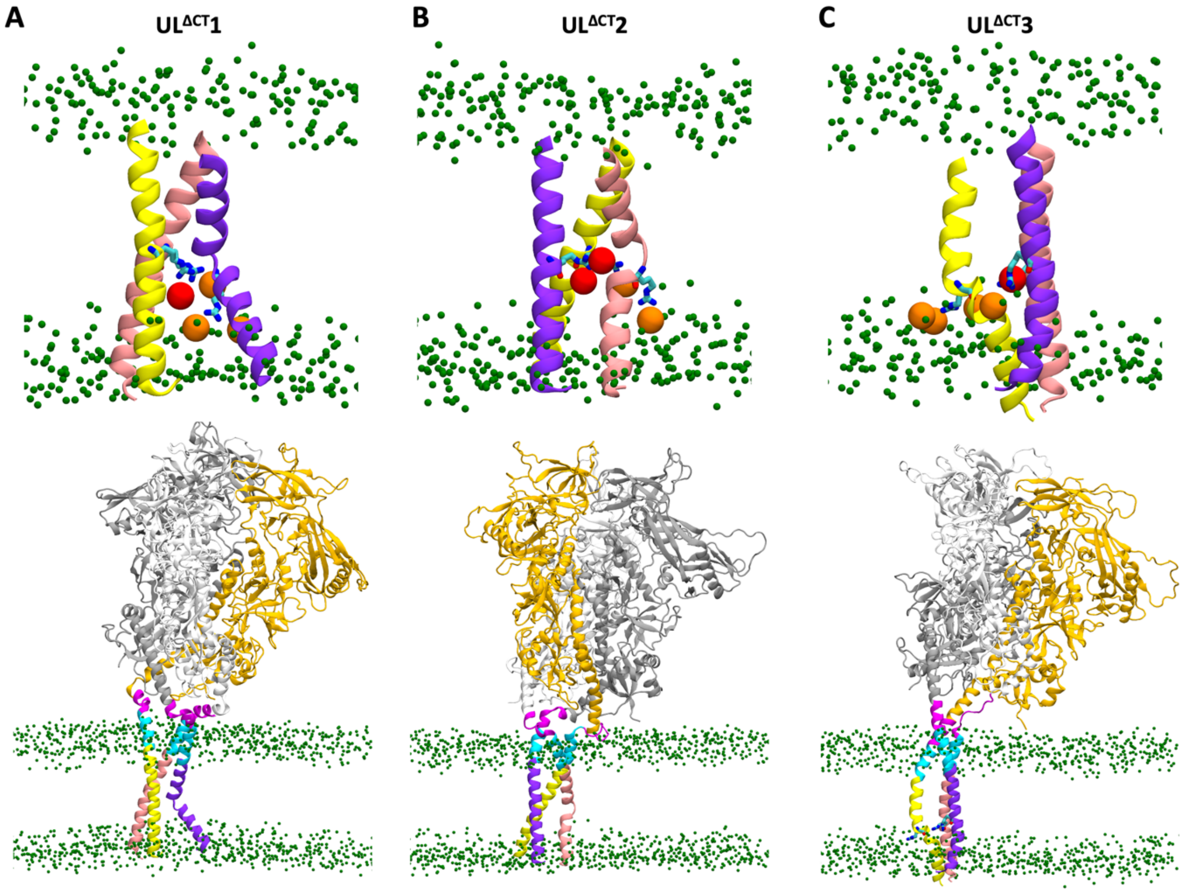

Figure S11. Local conformations of the TMD and global conformations of protein and membrane ($UL^{ΔCT}$ systems). Labeling and color coding are the same as in **Figure S8**.

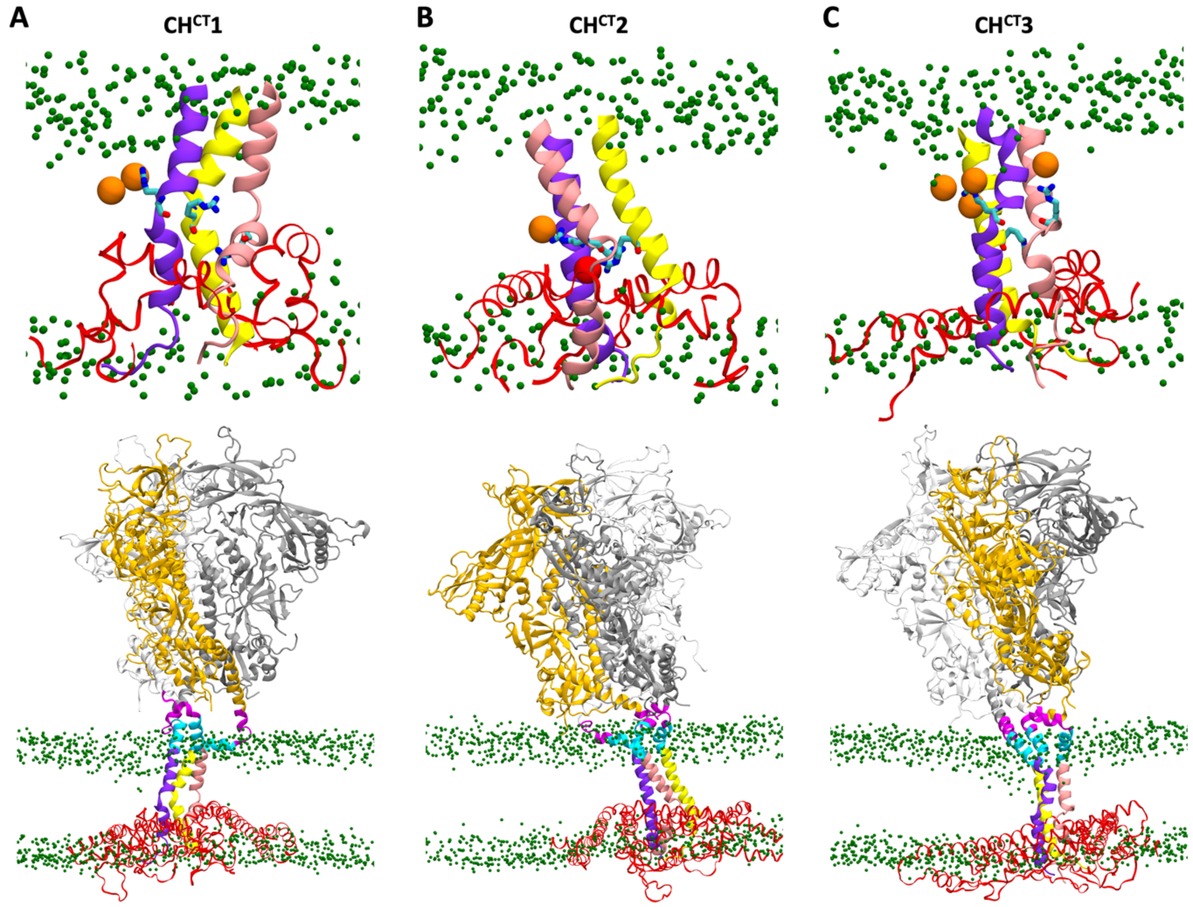

Figure S12. Local conformations of the TMD and global conformations of protein and membrane (CH^{CT} systems). Labeling and color coding are the same as in **Figure S8**, with the CT additionally shown in red.

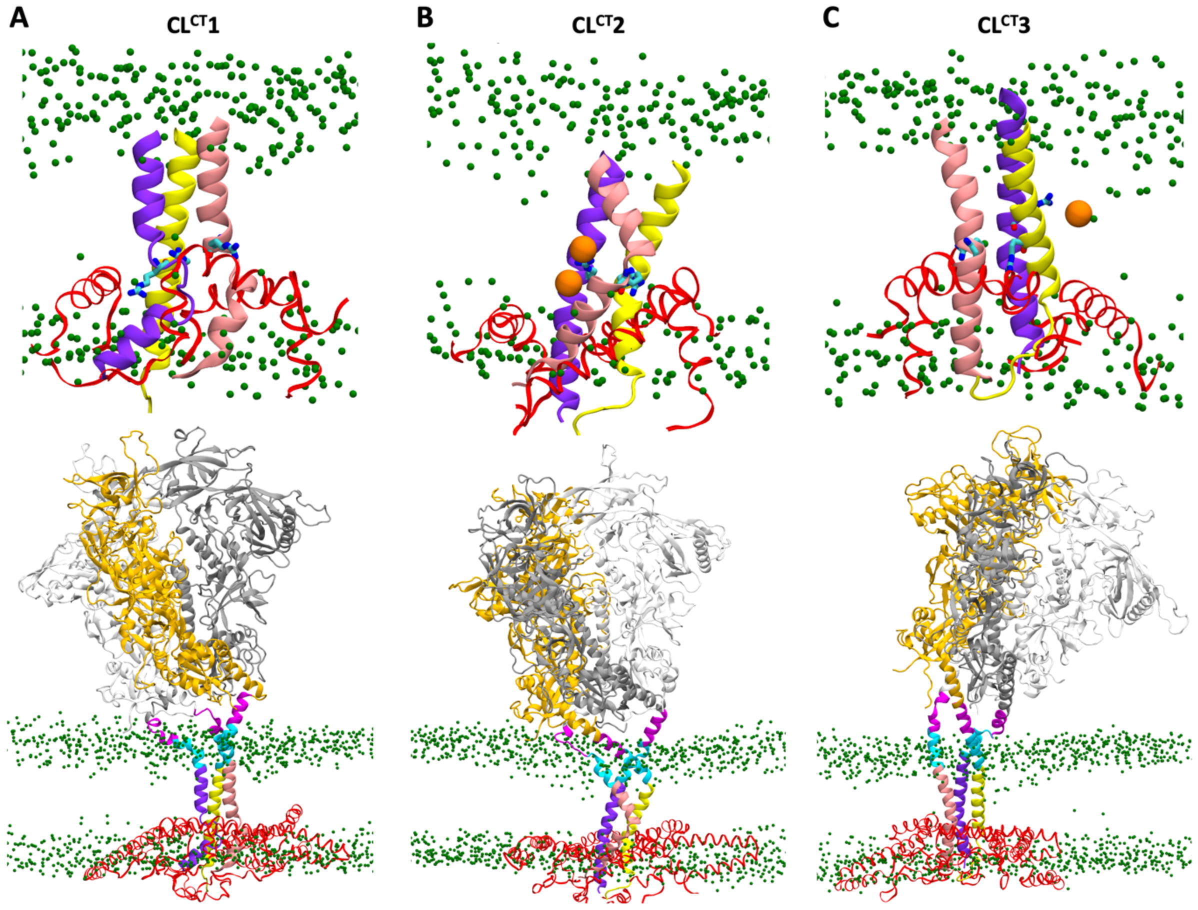

Figure S13. Local conformation of the TMD and global conformation of protein and membrane (CL^{CT} systems). Labeling and color coding are the same as in **Figure S12**.

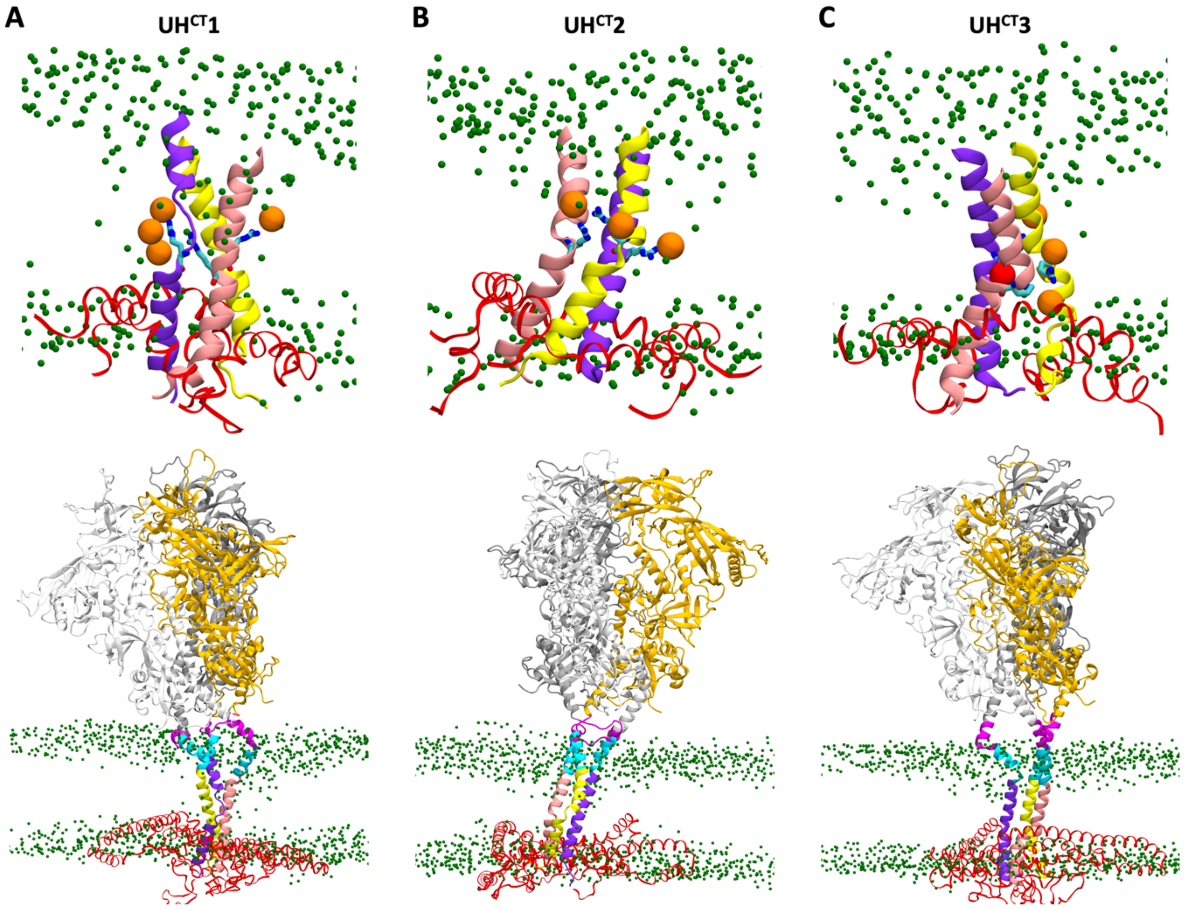

Figure S14. Local conformations of the TMD and global conformations of protein and membrane (UH^{CT} systems). Labeling and color coding are the same as in **Figure S12**.

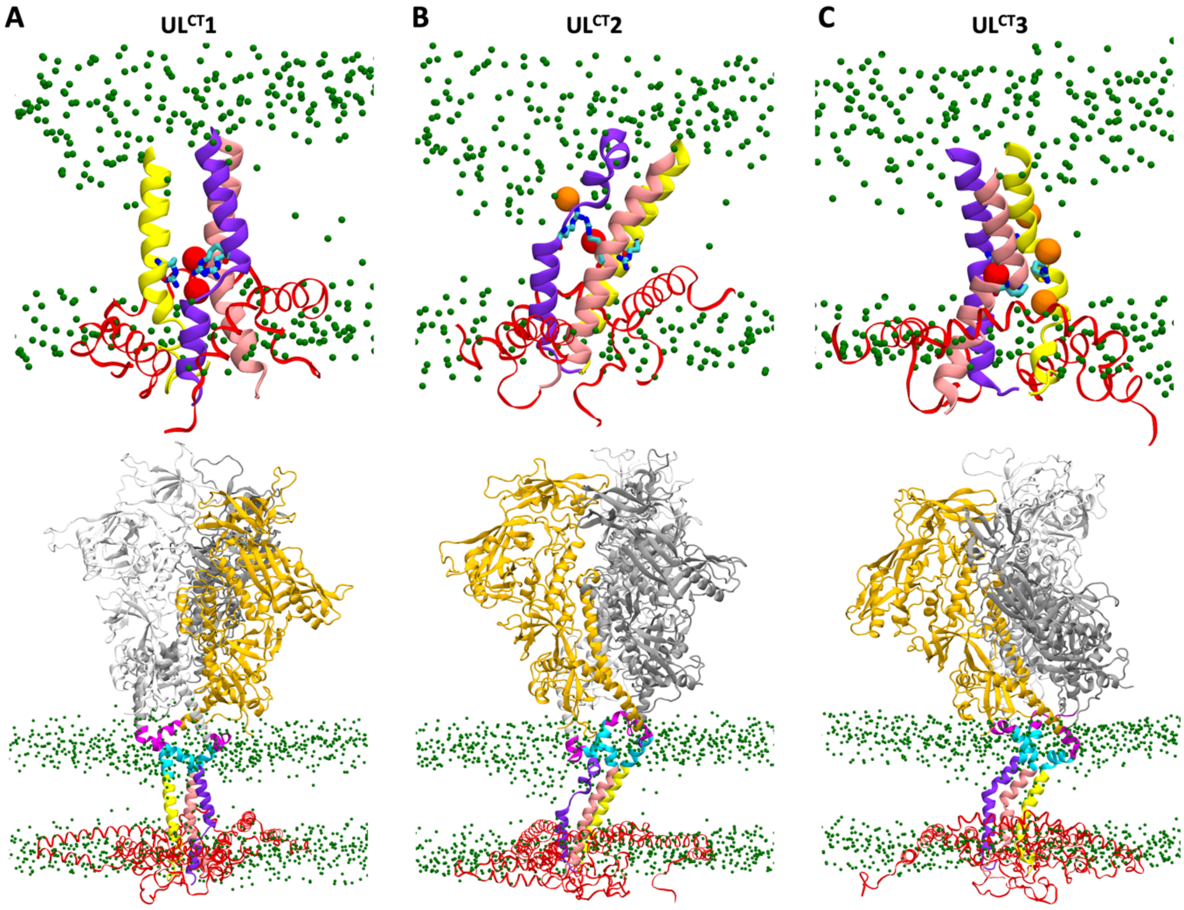

Figure S15. Local conformations of the TMD and global conformations of protein and membrane (UL^{CT} systems). Labeling and color coding are the same as in **Figure S12**.

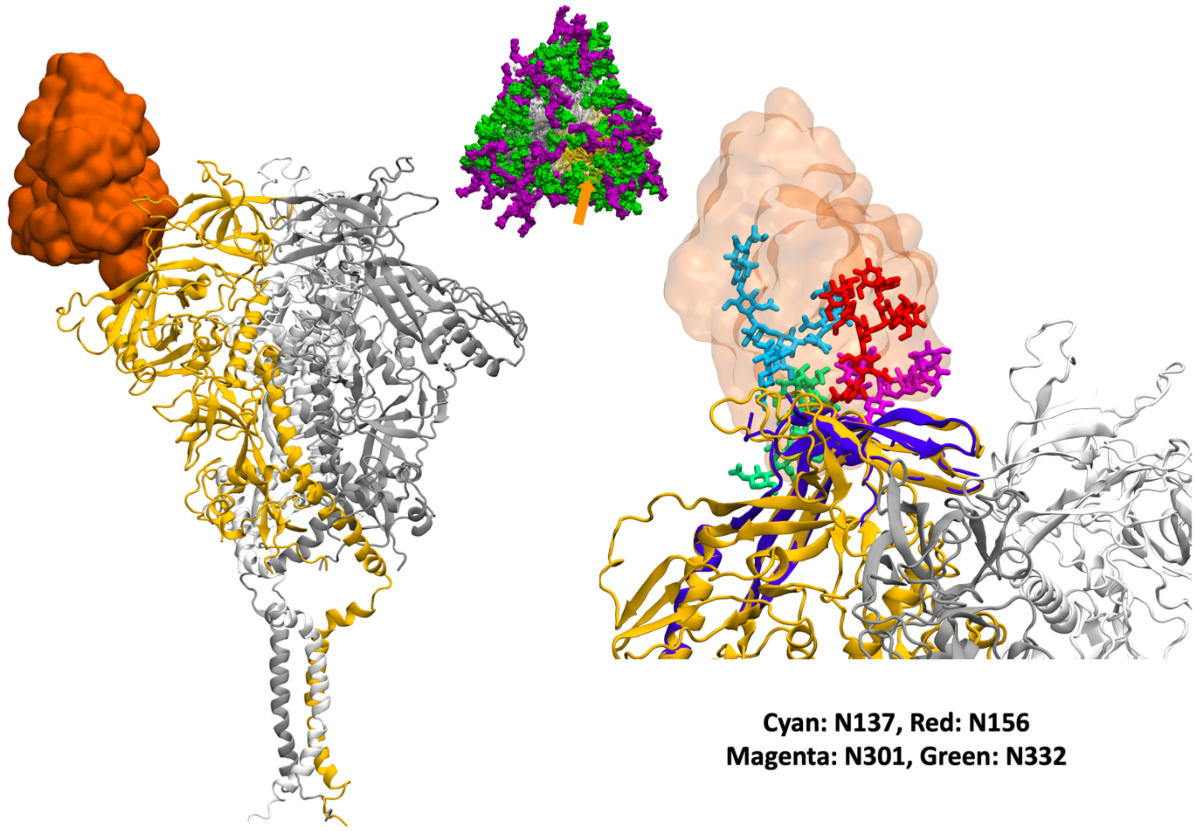

Figure S16. Shielding of antibody PGT128 epitope. (Left) Variable domains of the heavy and light chains aligned onto our modeled structure. (Middle) Top view of the glycosylated trimeric protein, with the orange arrow indicating the epitope location. (Right) Part of the epitope in the antibody-epitope complex PDB structure was used for structural alignment and is highlighted in purple. Glycans capable of hindering antibody binding are shown in various colors.

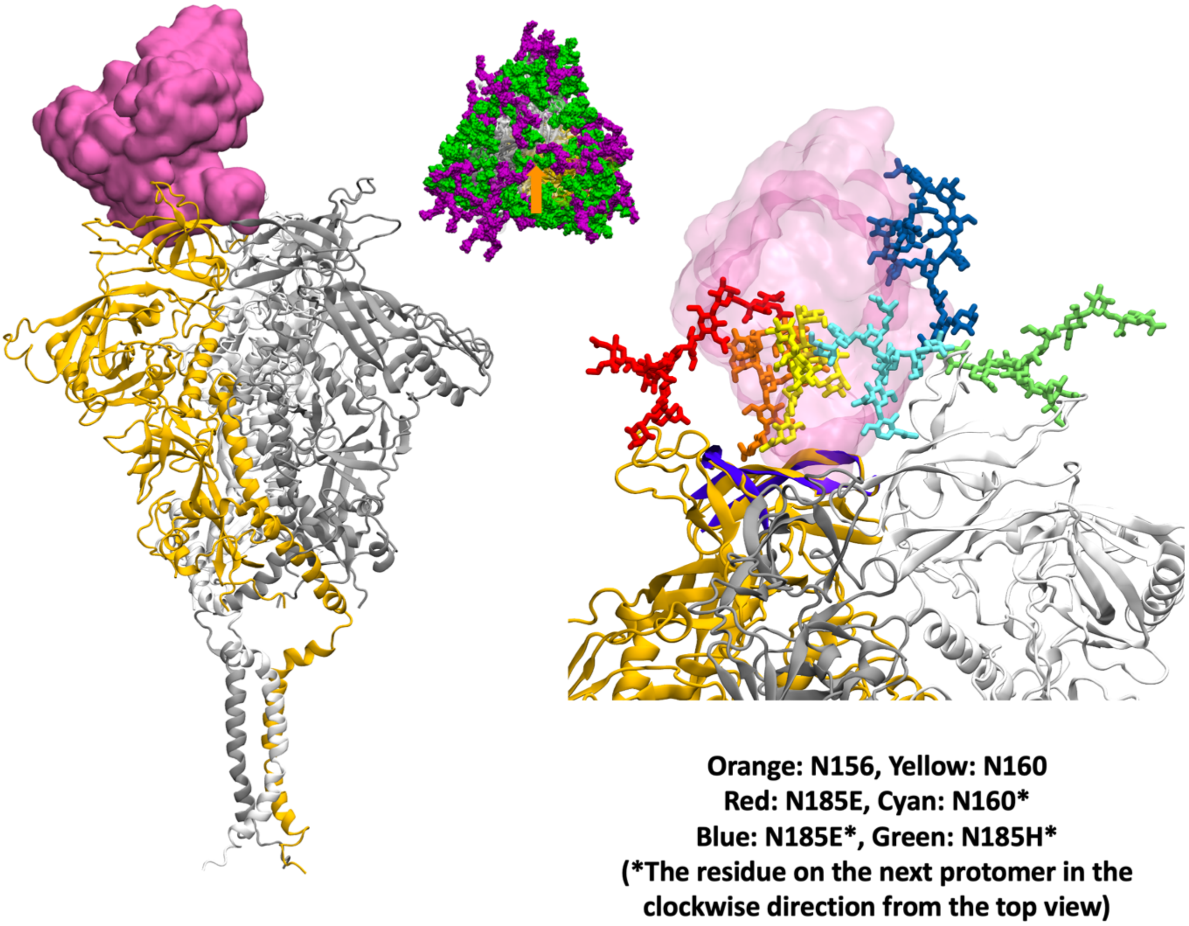

Figure S17. Shielding of antibody PG9 epitope. Labeling and color coding are the same as in **Figure S16**.

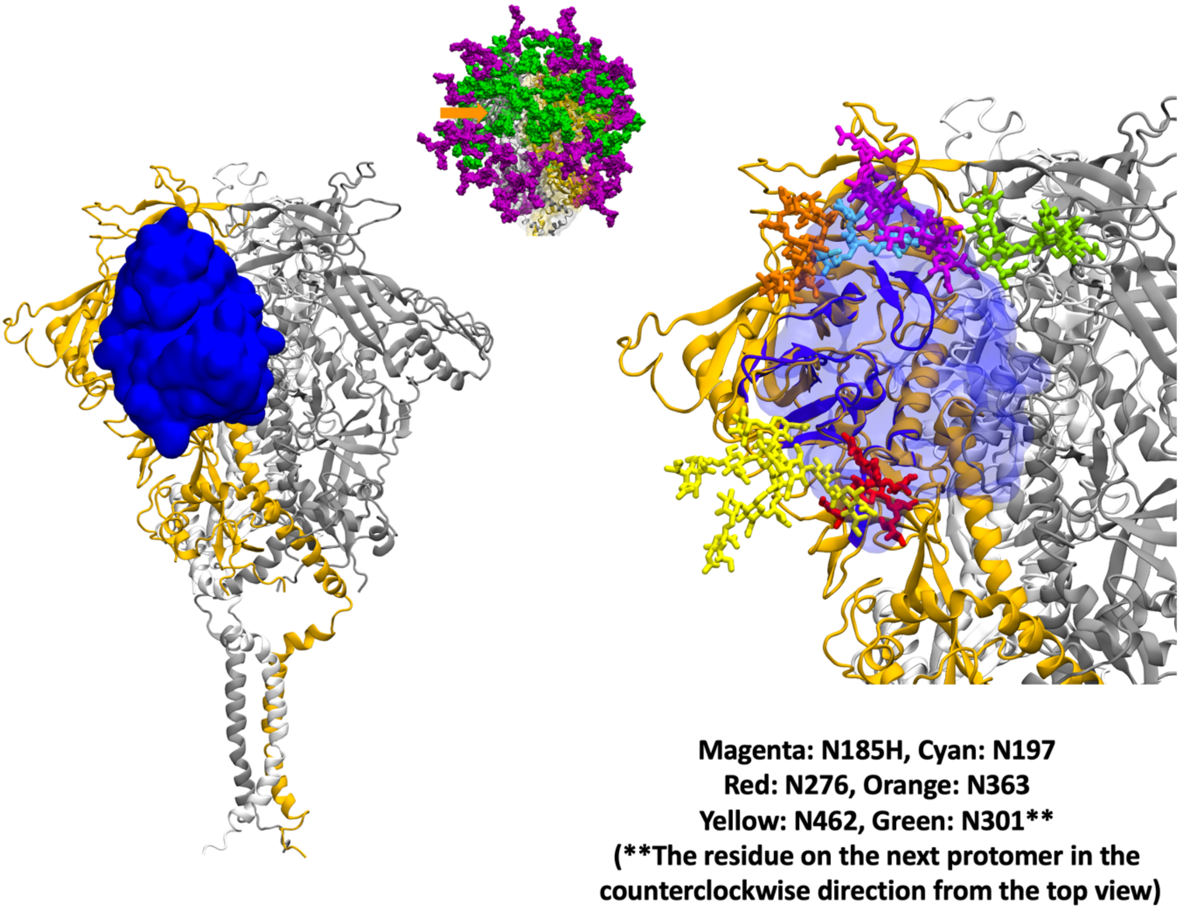

Figure S18. Shielding of antibody VRC01 epitope. Labeling and color coding are the same as in **Figure S16**. (Middle) The glycosylated trimeric protein is shown in a side view instead of the top view.

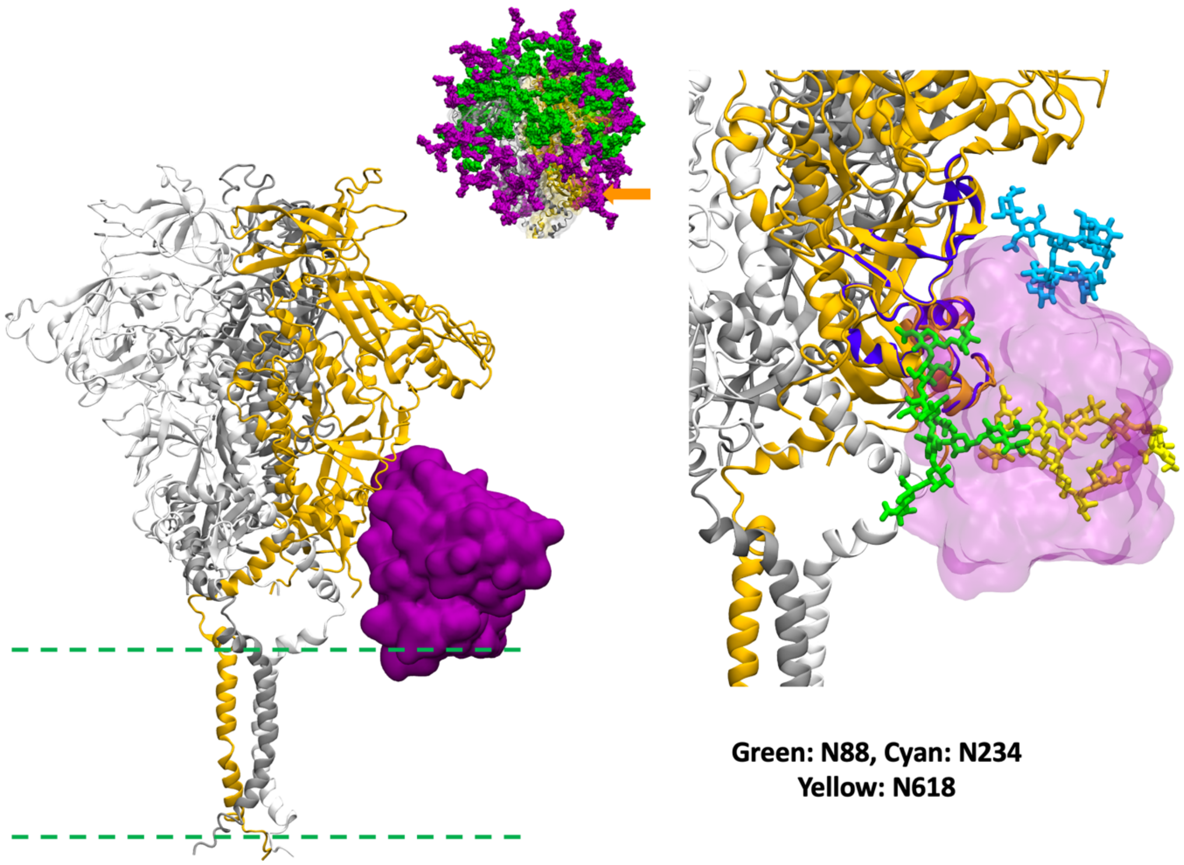

Figure S19. Shielding of antibody 35O22 epitope. Labeling and color coding are the same as in **Figure S18**. (Left) The dashed greens indicate the approximate location of the lipid headgroups.

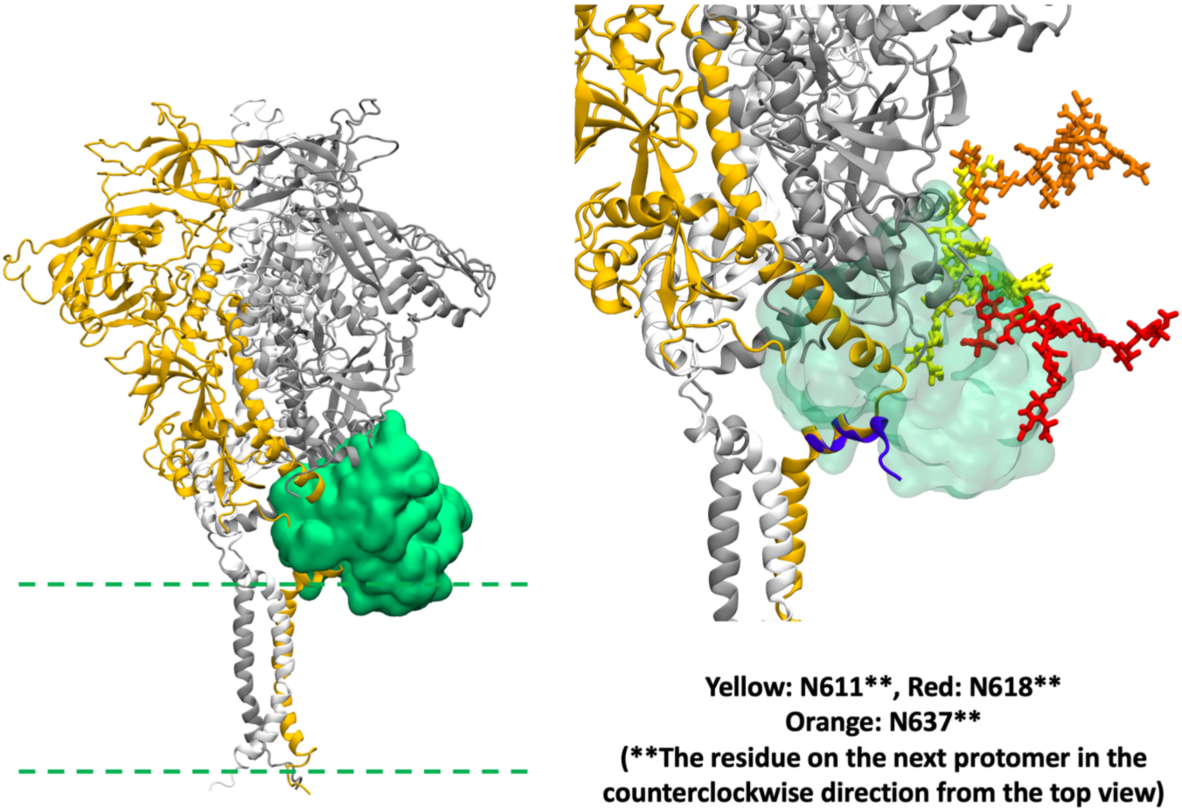

Figure S20. Shielding of antibody 4E10 epitope. Labeling and color coding are the same as in **Figure S19**.

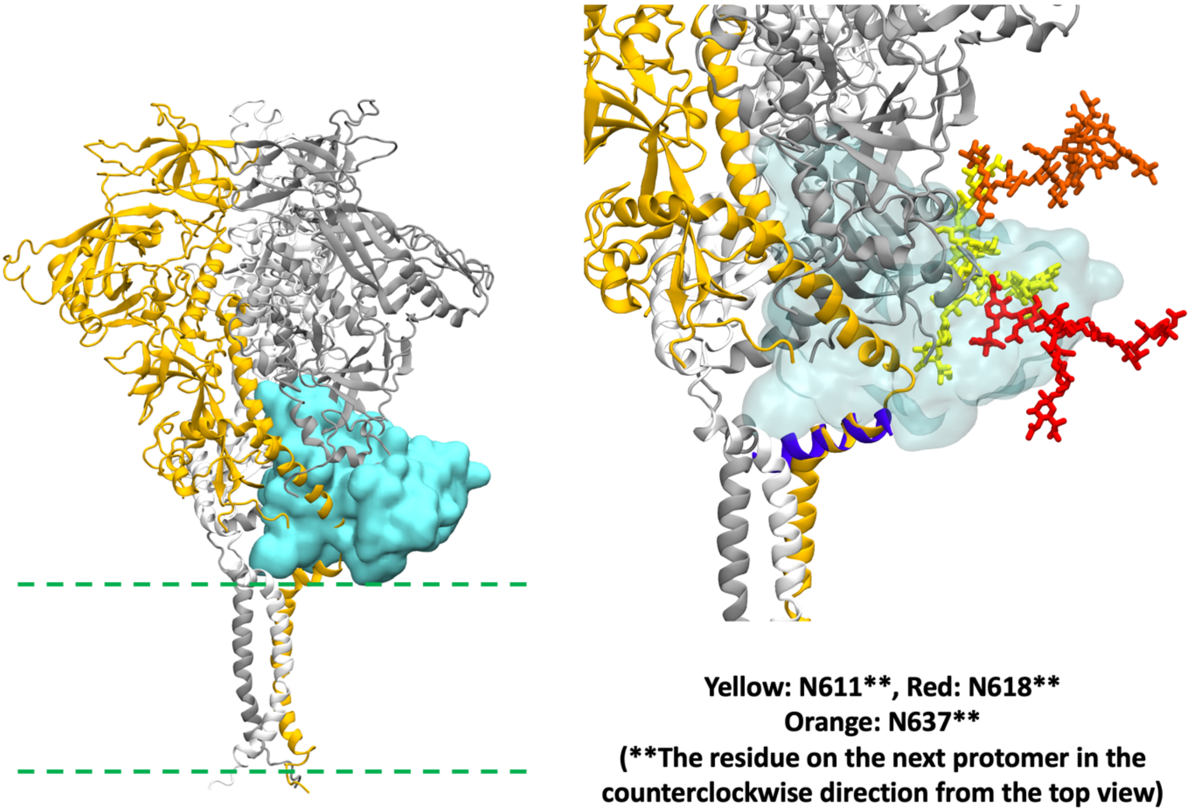

Figure S21. Shielding of antibody 10E8 epitope. Labeling and color coding are the same as in **Figure S19**.

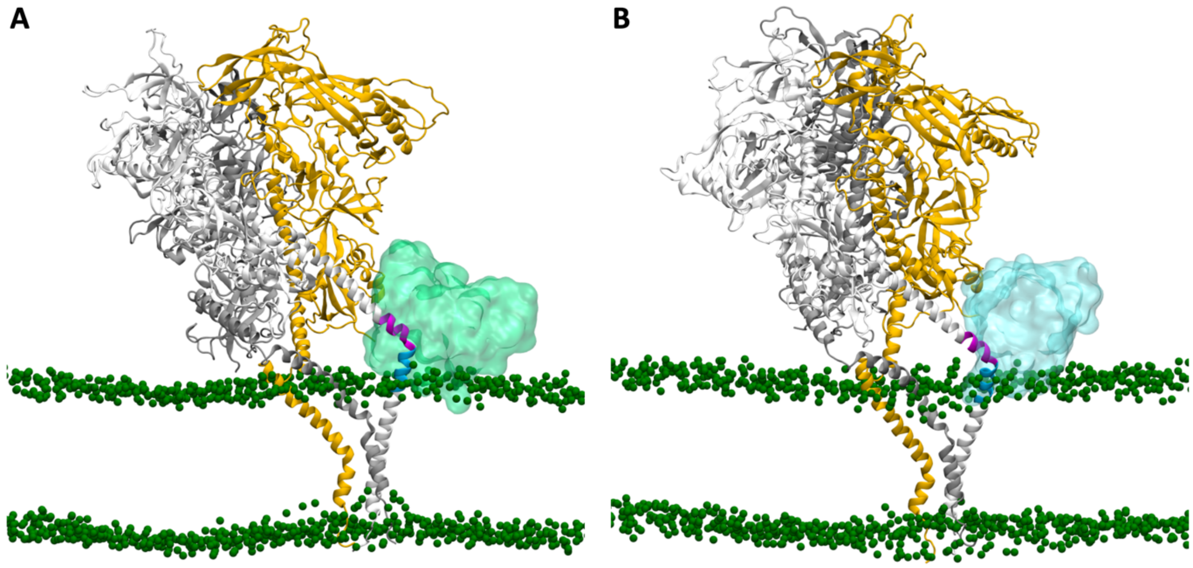

Figure S22. Snapshots showing the MPER of one protomer accessible to either 4E10 or 10E8. (A) Snapshot in which the MPER of the white protomer is accessible to 4E10 (cyan transparent surface) but not to 10E8. (B) Snapshot in which the MPER of the white protomer is accessible to 10E8 (green transparent surface) but not to 4E10.

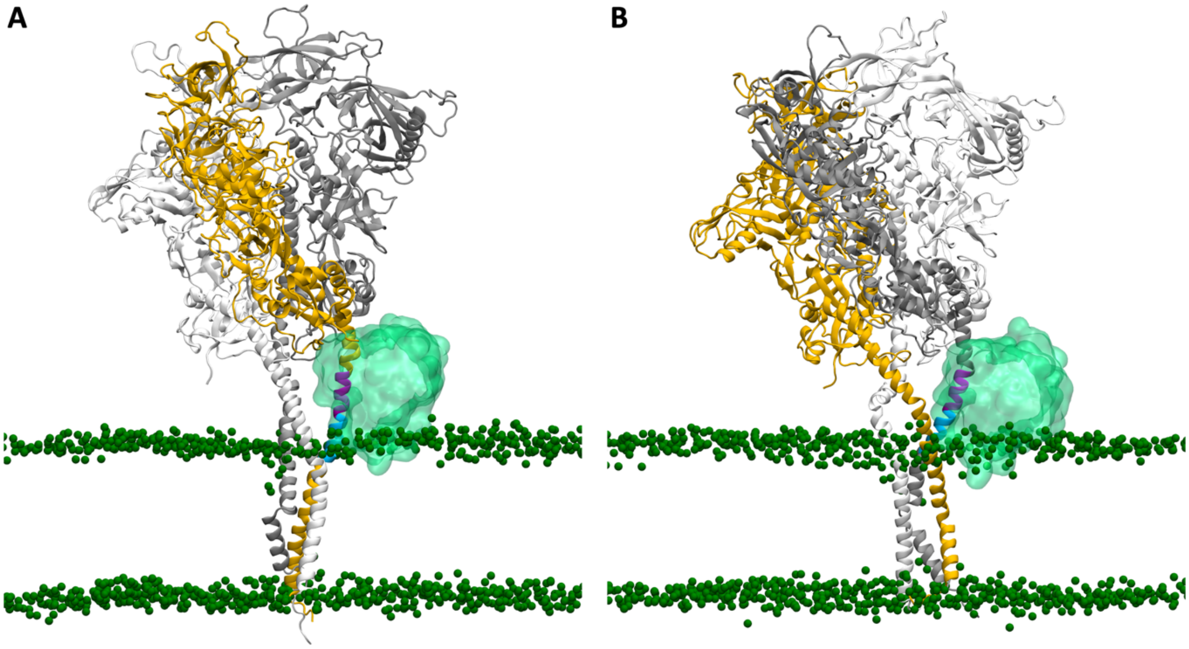

Figure S23. Snapshots showing the MPER of two protomers are accessible to 4E10. The MPER is accessible to 4E10, (A) exclusively in the yellow protomer, and (B) exclusively in the gray protomer.

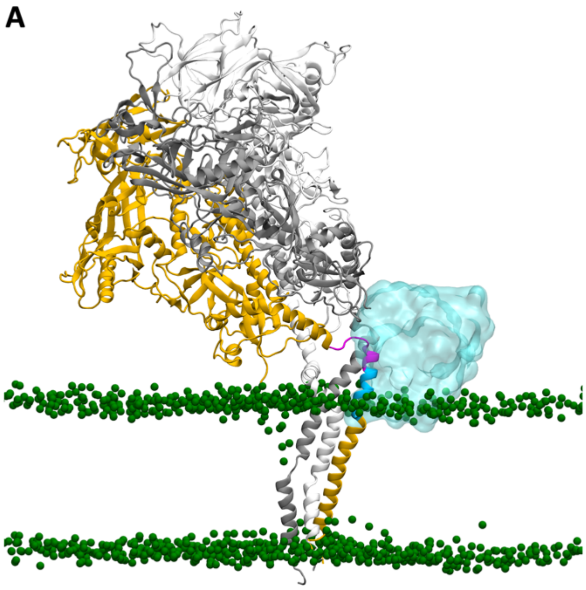

Figure S24. Snapshots showing the MPER of two protomers are accessible to 10E8. The MPER is accessible to 4E10, (A) exclusively in the yellow protomer, and (B) exclusively in the white protomer.

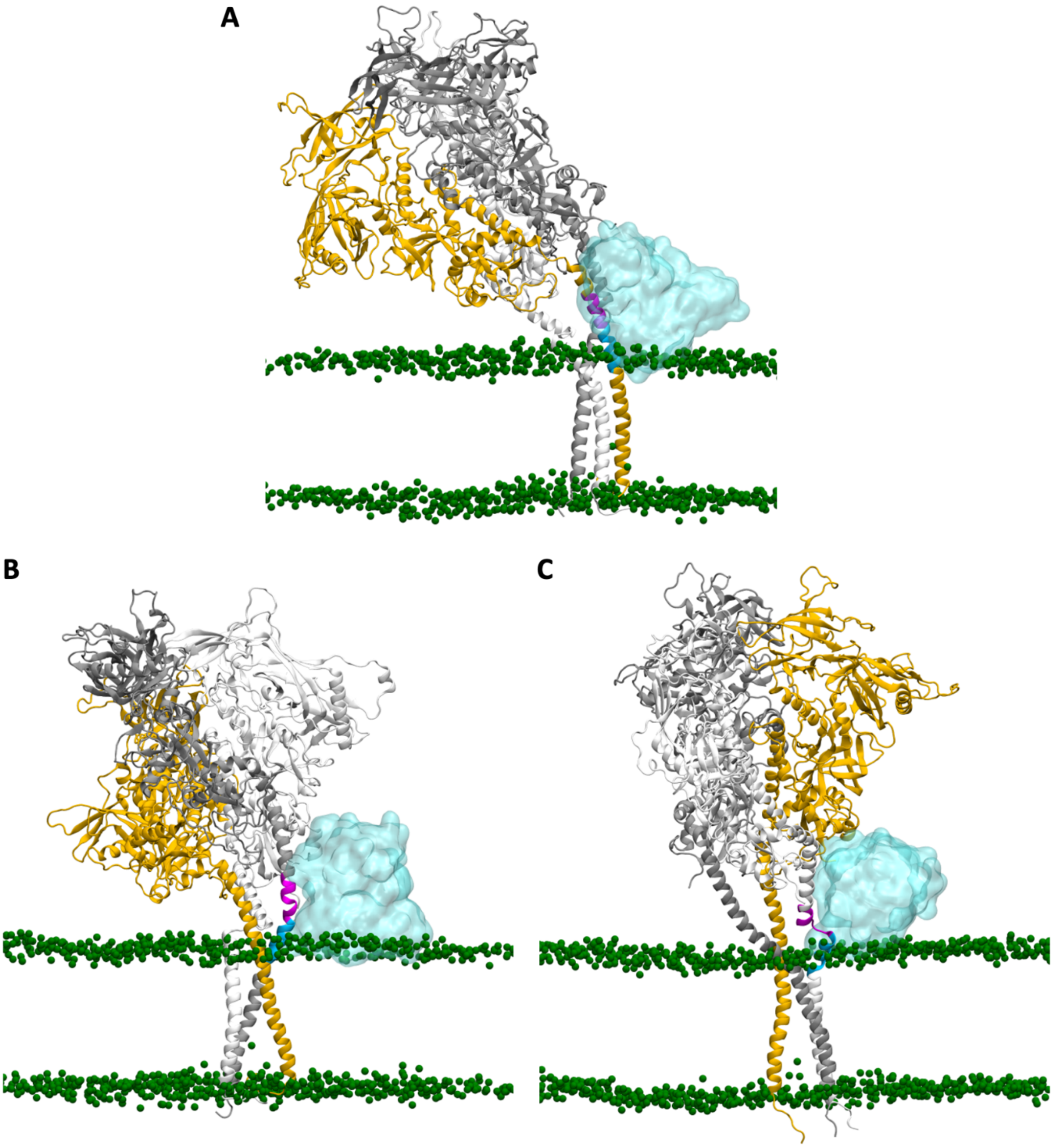

Figure S25. Snapshots showing the MPER of three protomers are accessible to 10E8. The MPER is accessible to 4E10, (A) exclusively in the yellow protomer, (B) exclusively in the gray protomer, and (C) exclusively in the white protomer.

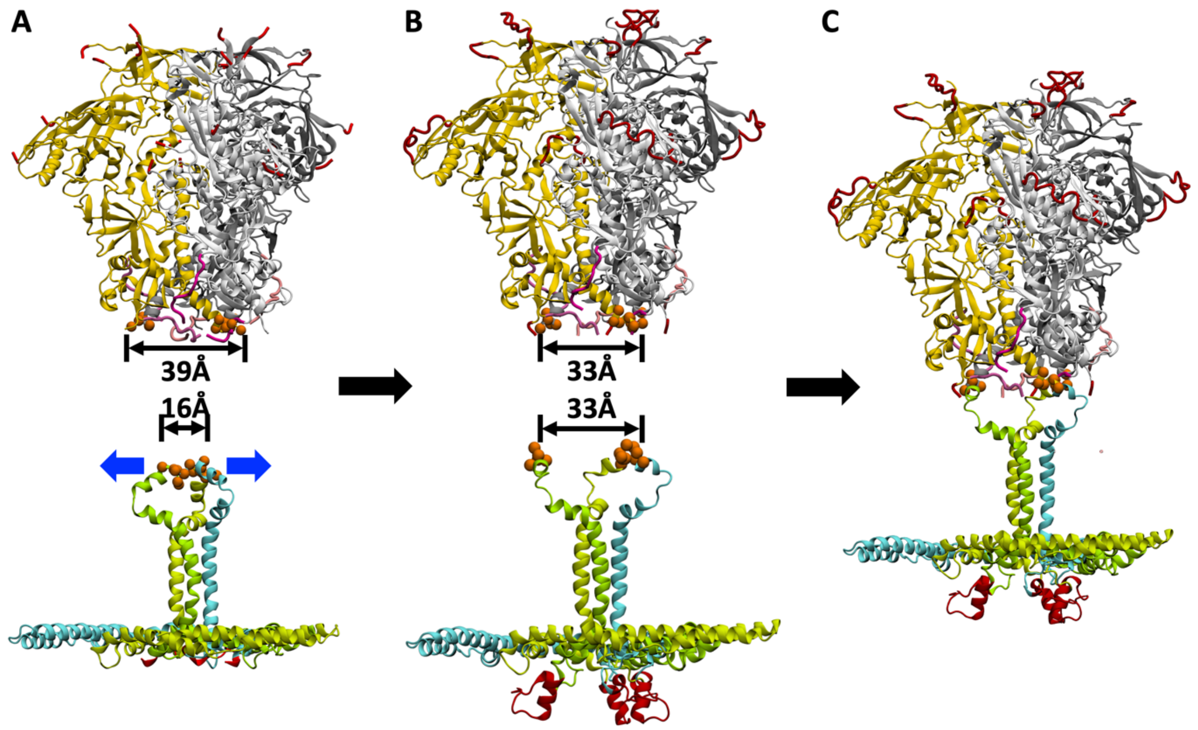

Figure S26. Steps in combining the structure of the ectodomain with the remaining domains. (A) The original PDB structures (PDB IDs: 6B0N and 7LOI) with the common residues highlighted by orange spheres. The distance between the Cα of D664 is 39 Å in 6B0N and 16 Å in 7LOI. The broken ends of missing loops are shown in red, and the cleavage site in magenta. (B) Missing loops were grafted from a modeled structure generated by I-TASSER (red). The extra Gly and Ser residues from the 2xG_4S linker introduced at the cleavage site in 6B0N were removed (magenta), and MD simulations were performed to adjust distances between the common residues in two PDB structures. (C) The common residues were aligned, and two structures were merged.

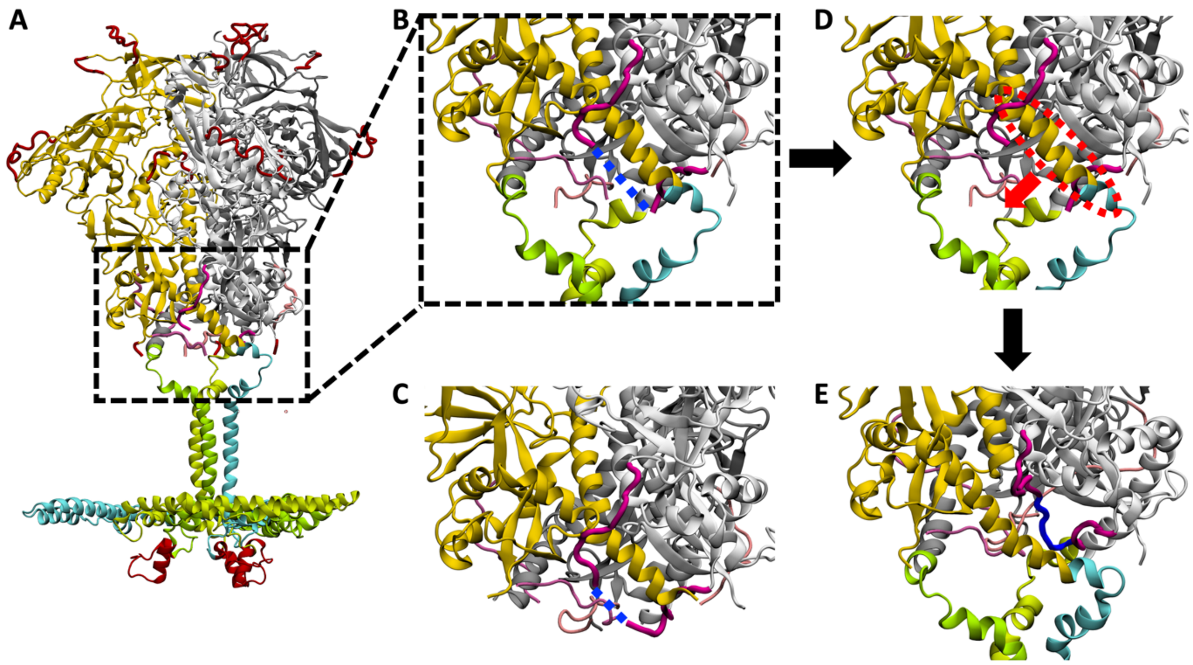

Figure S27. Steps in building the uncleaved model. (A) The cleaved model. (B) The cleavage site with the flanking loops in one promoter highlighted in dark magenta. If the flanking loops are connected along the blue dashed line, the protomer shown in yellow and cyan passes through the loop formed by the neighboring protomer in white, resulting in an entangled, knot-like topology. (C) The gap between the flanking loops is shorter in PDB structure 6B0N due to the presence of a 10-residue linker ^{508}GG<u>GGSGGGG</u>S^{511}, where the underlined residues are missing. (D) MD simulations were performed to adjust the length of the gap between the flanking loops to match that of the ^{508}REKR^{511} cleavage site, and to reposition the flanking loops and the HR2 helix (red circle) to prevent the connected loop from forming a knot-like structure with the neighboring protomer. (E) ^{508}REKR^{511} (blue) was modeled to connect the flanking loops.
